# Bisabolane Sesquiterpenes with Anti-Chlamydial Activity Isolated from *Ligularia narynensis*

**DOI:** 10.3390/ijms26031388

**Published:** 2025-02-06

**Authors:** Na Gao, Yi-Lin He, Hui-Ming Qi, Hong-Ying Yang, Guo-Li Li, Zhao-Cai Li, Tong Shen

**Affiliations:** 1 Research Institute, School of Chemistry and Chemical Engineering, Lanzhou Jiaotong University, Lanzhou 730070, China; gaonn08@163.com (N.G.); heyl@lzjtu.edu.cn (Y.-L.H.); qihmchem@163.com (H.-M.Q.); yanghy@lzjtu.edu.cn (H.-Y.Y.); ligl@lzjtu.edu.cn (G.-L.L.); 2 State Key Laboratory for Animal Disease Control and Prevention, Lanzhou Veterinary Research Institute, Chinese Academy of Agricultural Sciences, Lanzhou 730046, China

**Keywords:** *Ligularia narynensis*, bisabolane sesquiterpenes, anti-chlamydial activity

## Abstract

*Chlamydia* are obligate intracellular bacterial pathogens affecting humans and animals, causing miscarriage, stillbirth, or weak fetuses in the late stages of pregnancy of goats and sheep. Because there is no commercial vaccine for chlamydia in animals, drug treatment has become the most effective curative method. Natural products, also known as secondary metabolites, are becoming one of the main sources used in new drug development because of their structural diversity and biodiversity. In natural products, plant sources play a major role in the development process of new drugs. In this study, five undescribed highly oxygenated bisabolane sesquiterpenes (Pararubin W, Pararubin X, Pararubin Y., Pararubin Z, and Pararubin AA) were isolated from whole plants of *Ligularia narynensis*. Their chemical structures were determined via analyses of HRESIMS, IR, 1D, and 2D NMR data, along with the assignment of their relative configurations. These compounds were tested for their anti-chlamydial activity. The results show that compounds **1** and **5** inhibited the growth of *Chlamydia abortus* in host cells in a dose-dependent manner.

## 1. Introduction

The genus *Ligularia* belongs to the Asteraceae family, which includes about 150 species distributed in Europe and from the Himalayan region to Japan. In addition, about 111 species grow in China and are widely distributed from the southwest to the northeast [1]. Many of these plants have long been used as traditional or folk medicine for the treatment of scarlet fever [2], cough [3], chronic hepatitis B [4], tuberculosis [5], hemoptysis [4], and inflammation [6]. In past studies, various phytochemicals from the genus *Ligularia* were reported, including sesquiterpenes [7], monoterpene lactones [8], diterpenes [9], triterpenes [10], flavonoids [11], alkaloids [12], lignans [13], benzofurans [14], and sterols [15]. Among them, sesquiterpenes are the most important bioactive constituents, especially bisabolane sesquiterpenes, which have antibacterial [16], anti-inflammatory [17], and cytotoxic activities [18]. *Chlamydia* are obligate intracellular bacterial pathogens affecting humans and animals, causing miscarriage, stillbirth, or weak fetuses in the late stages of pregnancy of goats and sheep [19]. Because there is no commercial vaccine for chlamydia in animals, drug treatment has become the most effective curative method. This study investigated the constituents of *Ligularia narynensis* to search for anti-chlamydial components.

## 2. Results

### 2.1. Structural Elucidation

**Figure 1:**
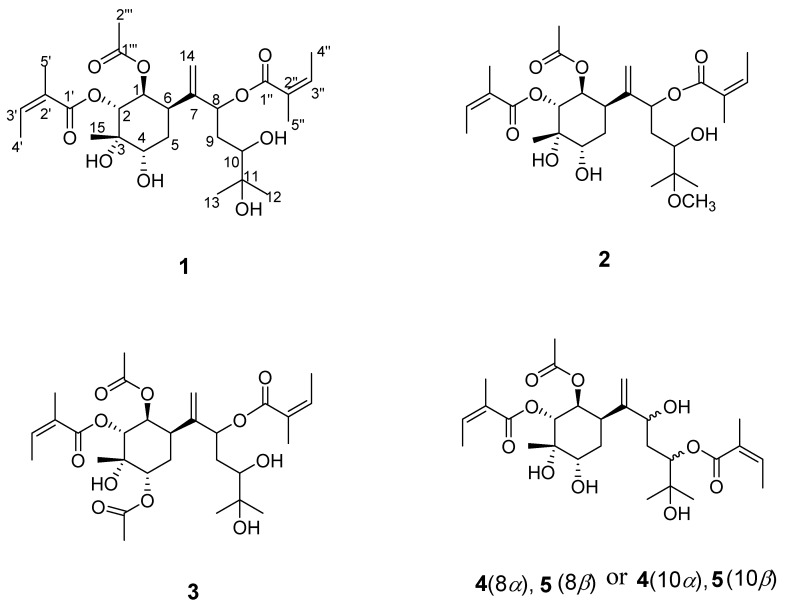
Structures of compounds **1**–**5**.

**Figure 2:**
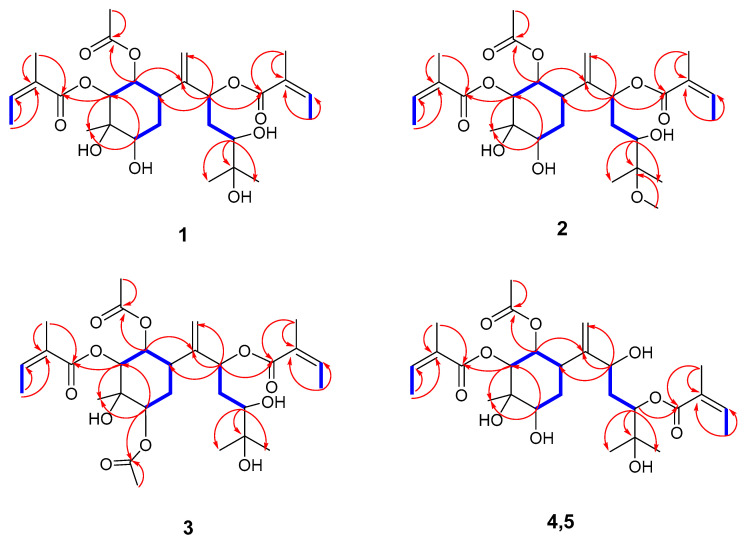
^1^H−^1^H COSY (blue bold) and key HMBC (red arrows) correlations of compounds **1**–**5**.

Compound **1** had the molecular formula C_27_H_42_O_10_ (Figure 1), indicating seven degrees of hydrogen deficiency based on data from ^13^C NMR and HRESIMS (*m*/*z* 549.2673, [M +Na]^+^) (calculated for C_27_H_42_O_10_Na, 549.2670). The IR spectrum showed that the compound contained functional groups such as hydroxy (3468 cm^−1^), carbonyl (1751 cm^−1^), and alkenyl (1457 cm^−1^). The ^1^H NMR spectra (Table 1) revealed a terminal double bond at *δ*_H_ (5.11 br s and 5.29 br s) and three methyl groups at *δ*_H_ (1.15 s, 1.17 s, and 1.32 s). The ^13^C and HSQC NMR (Table 2) spectra showed two angeloyl group signals at *δ*_C_ (168.7, 129.1, 139.7, 16.2, 20.9; 168.1, 128.8, 139.5, 16.0, and 20.6), an acetyl group signals at *δ*_C_ (171.8 and 21.0), and seven oxygenated carbons signals at *δ*_C_ (75.8, 75.4, 73.4, 74.4, 72.3, 71.5, and 66.6), including five methine groups and two quaternary carbons. There were also eight carbons signals, including two methylene signals at *δ*_C_ (31.4 and 37.2), one methine at *δ*_C_ (36.4), a terminal double bond (148.2, 115.8), and three methyl carbons. According to the four spin-coupling systems of H-2/H-1/H-6/H_2_-5/H-4, H-8/H_2_-9/H-10, H_3_-4″/H-3″, and H_3_-4′/H-3′ in ^1^H−^1^H COSY, the planar configuration of compound **1** was further determined via the following HMBC correlations (Figure 2): from H-1 to C-2, C-3, C-5, C-6, C-7, and C-1‴; from H-2 to C-1, C-3, C-4, C-6, C-15, and C-1′; from H-4 to C-2, C-3, C-5, C-6, and C-15; from H-8 to C-7, C-9, and C-10; from H-4′ to C-3′ and C-2′; from H-4″ to C-3″ and C-2″; from H-5″ to C-1″ and C-2″; from H-5′ to C-1′ and C-2′; from H-2‴ to C-1‴; and from H-10 to C-11, C-12, and C-13. Contrary to the typical sesquiterpene skeleton of bisabolanes, the hydroxyl group at C-5 disappeared.

**Figure 3:**
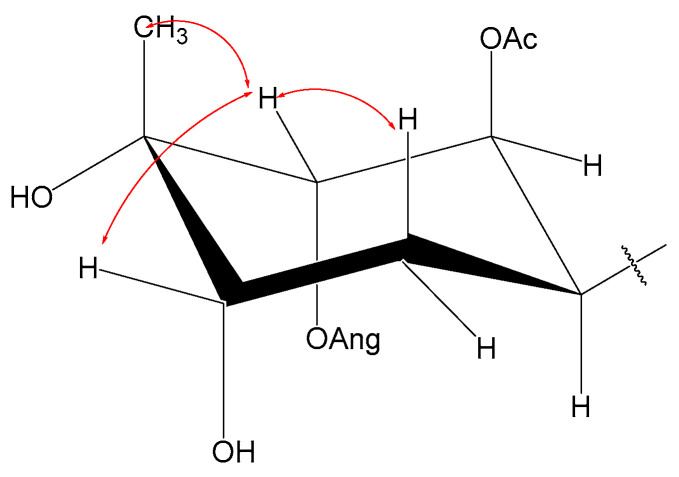
NOESY (red double arrow) correlations of compound **1**.

The relative configuration of **1** was determined using the ^1^H NMR coupling constants: the small coupling constants of H-1/H-6 (*J*_1,6_ = 3.0 Hz) and (*J*_1,2_ = 3.6 Hz) demonstrated that H-1 should have been *α*-axial and H-2 must have been *β*-equatorial, considering H-6 as *α*-oriented. The H-15 and H-4 configurations were determined to be *β*-oriented based on the key NOESY correlations of H-2/H-15 and H-15/H-4, as shown in Figure 2. The NOESY correlations (Figure 3) were in accordance with the above observations. Thus, the structure of **1** was finally identified and named Pararubin W.

Compound **2** was assigned the molecular formula C_28_H_44_O_10_ from the HRESIMS ion peak at *m*/*z* 541.3002 [M + H]^+^ (calculated for C_28_H_45_O_10_, 541.3007). Its IR spectrum displayed absorption bands of hydroxy (3467 cm^−1^), carbonyl (1751 cm^−1^), and alkenyl (1447 cm^−1^) groups. The NMR data (Table 1 and Table 2) highly resembled those of **1,** except for a methyl signal (*δ*_H_ 3.22 (s, 3H), *δ*_C_ 49.6)). The ^13^C NMR chemical shifts in C-11 (*δ*_C_ 73.4) in **1** changed to (*δ*_C_ 78.2) in **2**, indicating that an oxo methyl group in **2** at C-11 replaced a hydroxy group in **1**. The location was confirmed by analyzing the HMBC correlation from H-OCH_3_ to C-11. The relative configuration of **2** was deduced to be the same as that of **1** (Figure 3) via the NOESY correlations and an analysis of the vicinal coupling constant values. Therefore, compound **2** was determined as shown and named Pararubin X.

Compound **3** was assigned a molecular formula of C_29_H_44_O_11_ via HRESIMS at *m*/*z* 591.2780 [M + Na]^+^ (calculated for C_29_H_44_O_11_Na, 591.2776), still retaining seven degrees of unsaturation. Its IR absorption displayed the presence of hydroxy (3484 cm^−1^), carbonyl (1721 cm^−1^), and alkenyl (1648 cm^−1^) moieties. The NMR data shared high similarity with those of **1**. However, the hydroxy group at C-4 in **1** was acetylated in **3** (*δ*_C_ 172.0, 21.1), which was further confirmed by the HMBC correlations of OAc-4/C-4. Accordingly, the relative configuration of **3** was determined to be identical to that of **1**. Consequently, the structure of **3** was defined and named Pararubin Y.

Compound **4** had a molecular formula of C_27_H_42_O_10_, according to HRESIMS at *m*/*z* 549.2675 [M + Na]^+^ (calculated for C_27_H_42_O_10_Na, 549.2670). The IR spectrum of **4** showed absorption bands characteristic of hydroxyl (3433 cm^−1^), carbonyl (1718 cm^−1^), and alkenyl (1595 cm^−1^) groups. Similar NMR data (Table 1 and Table 2) showed that the carbon skeleton of **4** was the same as that of **1**. The NMR data showed, for **4**, a carbon skeleton nearly identical to that of **1**, with the only difference being an angeloyloxy group, which was confirmed by the ^13^C NMR chemical shifts in C-8 (*δ*_C_ 75.8) and C-10 (*δ*_C_ 75.4) in **1** changing to C-8 (*δ*_C_ 71.6) and C-10 (*δ*_C_ 77.6) in **4**, and the ^1^H NMR signals at *δ*_H_ 5.59 (1H, dd, *J* = 11.0, 2.5 Hz, H-8) and *δ*_H_ 3.36 (1H, dd, *J* = 10.7, 1.7 Hz, H-10) in **1** changing to *δ*_H_ 3.97 (1H, dd, *J* = 10.3, 2.8 Hz, H-8) and *δ*_H_ 5.20 (1H, dd, *J* = 10.3, 1.9 Hz, H-10) in **4**. This deduction was further verified by the HMBC correlations of H-1/C-2/C-3/C-5/C-6/C-7/C-8, C-8/C-10, H-4″/C-3″/C-2″/C-1″, and H-10/C-1″. The NOESY correlations showed that compounds **4** and **1** had a similar relative configuration (Figure 3) regarding the six-membered ring. However, the configuration of the sidechain changed when comparing the ^13^C NMR chemical shifts in C-7 (*δ*_C_ 151.6) and C-14 (*δ*_C_ 112.8) in **4** to C-7 (*δ*_C_ 148.2) and C-14 (*δ*_C_ 115.8) in **1,** which might have been related to stereochemistry at C-8 or Δ^7^. Following the above, the structure of 4 was defined and named Pararubin Z.

Compound **5** was obtained as a colorless gelatinous constituent. Its molecular formula was identical to that reported for **4**, based on the comparison of their ^13^C NMR data and HRESIMS. Their NMR data (Table 1 and Table 2) were highly similar to those of **4**, except for the ^13^C NMR chemical shifts in C-7 (*δ*_C_ 151.6), C-8 (*δ*_C_ 71.6), and C-14 (*δ*_C_ 112.8) in **4** changing to *δ*_C_ 149.4, *δ*_C_ 74.9, and *δ*_C_ 115.9 in **5** and the ^1^H NMR at H-10 (*δ*_H_ 5.20 dd (10.3, 1.7)) in **4** changing to *δ*_H_ 4.73 dd (overlap) in **5**. The H-10 resonance was shifted significantly up field (Δδ 0.47 ppm) in **5**. This suggested compounds **4** and **5** to be an epimeric pair at C-8 and/or C-10. Accordingly, compound **5** was determined as shown and named Pararubin AA.

While it was insufficient to conclude the determination of the absolute conformation of positions 8 and 10 of compounds **1**–**5** solely from the ECD. compounds **4** and **5** were determined to be an epimeric pair at C-8 and/or C-10, which was confirmed by the ECD spectra in SI Figure S41, indicating that 5 gave an exactly opposite cotton effect at 250 nm compared to **4**. The experimental ECD curve agreed well with compounds **1**, **2**, and **3**.

### 2.2. The Anti-Chlamydial Activity of the Compounds

*Chlamydia* spp. is a group of globally distributed bacterial pathogens affecting humans and animals. The screening of compounds for pharmaceutical value is an important strategy for the development of new drugs or treatment strategies against infectious diseases [20]. About 80% of the world relies on plant-based medicine to treat all ailments, while 70% of pharmaceutical therapeutics are based on natural ingredients. Using herbal medicines as the starting point, new anti-chlamydial drugs can surely be developed. In this regard, it should be noted that bisabolane-type sesquiterpenes were identified in Chinese herbal drugs and shown to display multiple activities such as antitumor [21], anti-inflammatory [22], and antibacterial [23] effects. Thus, in order to evaluate the medical potential of novel bisabolane-type sesquiterpenes **1** to **5**, isolated from the medicinal herb *L. narynensis*, the aim of the current study was to determine their anti-chlamydial activity.

**Figure 4:**
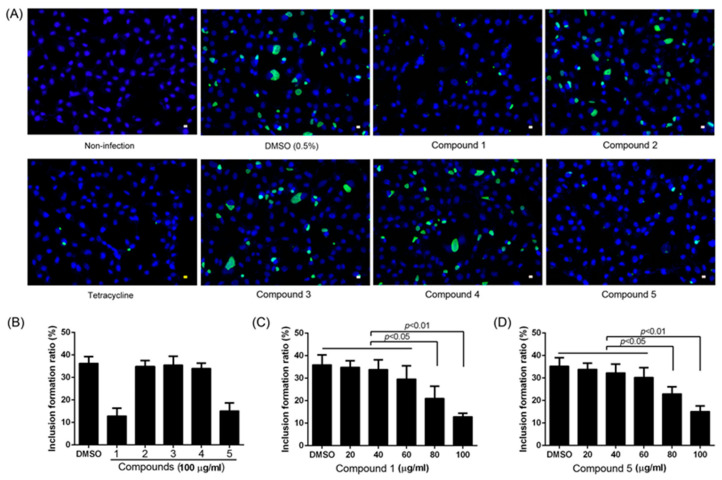
Anti-chlamydial effects of compounds **1**–**5**. Various concentrations of the compounds were applied to the *C. abortus* strain GN6 cultured in McCoy cells. Anti-chlamydial activity was represented by the inclusion formation ratio, based on immunofluorescent staining of *C. abortus* inclusions. (**A**) Observation of *C. abortus* inclusions in cell cultures under treatment with tetracycline (5 μM) as a positive control or the tested compounds at the concentration of 100 μg/mL. (**B**) *C. abortus* inclusion formation ratio in cell cultures treated with the tested compounds at 100 μg/mL final concentrations. (**C**) Treatment with compound **1** reduces the inclusion formation ratio of *C. abortus* in cell cultures. (**D**) Treatment with compound **5** reduces the inclusion formation ratio of *C. abortus* in the cell cultures. These data are the mean ± SD and representative of three independent experiments.

There are various mammalian hosts infected by *Chlamydia* species, which are obligate intracellular bacteria. Using *Chlamydia abortus*-infected McCoy cells as an experimental model, we tested the anti-chlamydial effects of compounds **1**–**5** at a concentration of 100 μg/mL. As the bacterium is sensitive to tetracycline, tetracycline at a 5 μM final concentration was used as a positive control. As a result, few and small intracellular inclusions of *C. abortus* could be observed in the tetracycline-treated cell cultures. Similarly, compounds **1** and **5** exhibited excellent anti-chlamydial effects, and chlamydial inclusions were markedly smaller and fewer in number (Figure 4A,B), while compounds **2**, **3**, and **4** did not affect the intracellular growth of the bacterium. Notably, compounds **1** and **5** inhibited the growth of *C. abortus* in the host McCoy cells in a dose-dependent manner. In addition, at a 80 µg/mL or higher concentration of compounds **1** and **5**, the formation of bacterial inclusions within McCoy cells was significantly reduced (Figure 4C,D). It is worth mentioning that all five compounds exhibited no cytotoxicity to McCoy cells when treated at a concentration of 100 µg/mL for 72 h.

## 3. Discussion

Five undescribed bisabolane sesquiterpenoids (**1**–**5**) were isolated from the extract of whole plants of *L. narynensis.* Their structures were determined via analyses of HRESIMS, IR, and NMR data. The results of the anti-chlamydial assays show that compounds **1** and **5** exhibited a significant anti-chlamydial effect on *Chlamydia abortus* in a dose-dependent manner. The preliminary structure–activity relationship was discussed according to the anti-chlamydial effect on *Chlamydia abortus* of these bisabolane sesquiterpenoids. Comparing the anti-chlamydial effect of the compounds (**4** vs. **5**), we showed that the stereochemistry at C-8 and/or C-10 of the compounds had a great influence on the activity. The compounds were less active when they presented a methoxy group at C-11 or an acetyloxy group at C-4 instead of an hydroxyl group (**1** vs. **2** and **1** vs. **3**). These two compounds are promising future agents for the treatment of chlamydia in the fight against the development of multidrug resistance. In the meantime, this study provides a more scientific basis for the ethnopharmacological uses of *L. narynensis* and the development of novel anti-chlamydia drugs.

## 4. Materials and Methods

### 4.1. General Experimental Procedures

Optical rotations were recorded using a Perkin-Elmer Model 341 polarimeter (PerkinElmer, Wellesley, MA, USA). The IR spectra were obtained with a Bruker TENSOR27 spectrometer with KBr disks (Bruker, Karlsruhe, Germany). The electronic circular dichroism (ECD) spectra were measured by the Olis DSM-1000 spectrometer (OLIS, Seattle, WA, USA), while the UV spectra were recorded on a Shimadzu UV-3600 Plus spectrophotometer (Shimadzu, Kyoto, Japan). Nuclear magnetic resonance (NMR) spectroscopic data were recorded on a Bruker 500 MHz AVANCE NEO spectrometer (Bruker, Karlsruhe, Germany). The chemical shifts (*δ*) were given relative to the TMS, and the *J* values were given in Hz. The HRESIMS data were derived through a Bruker Daltonics APEX II (*m*/*z*) mass spectrometer (Bruker, Karlsruhe, Germany). Semi-preparative HPLC was performed on a SHIMADZU LC-2030 series pump (Shimadzu, Kyoto, Japan), equipped with a photodiode array detector and an X Bridge BEH C18 OBD Prep Column (5 μm, 250 × 10 mm) (Waters, Milford, CT, USA). The compounds in the crude extracts were isolated and purified using macroporous resin (HP-20) (Mitsubishi, Tokyo, Japan) and silica (200−300 mesh) via column chromatography (Qingdao Marine Chemical Co., Qingdao, China). Fluorescent silica gel GF_254_ (Qingdao Marine Chemical Co., Qingdao, China) plates were used for TLC, and the spots were visualized under ultraviolet light at 254 nm or spraying with 5% H_2_SO_4_ alcohol solution, followed by heating.

### 4.2. Plant Material

Whole *Ligularia narynensis* O.Fedtsch. and B.Fedtsch plants were collected in July 2019 from the Xinjiang province (east longitude 42°38′, north latitude 43°15′), China, and identified by Yan Zhao, from the Xinjiang Academy of Agricultural Sciences. A sample (No. lzyw20190516) was deposited in the laboratory of the Institute of Natural Medicine Development, Lanzhou Jiaotong University.

### 4.3. Extraction and Isolation

Air-dried and powdered whole *L. narynensis* (3.6 kg) plants were extracted with 50 L MeOH (7 d/t, 3 times) at room temperature. The combined MeOH extract was concentrated under reduced pressure to yield a residue (328 g), which was suspended in H_2_O and extracted three times with petroleum ether, EtOAc, and *n*-BuOH. The EtOAc layer was concentrated under reduced pressure, and the crude extract (127 g) was separated by HP-20 macroporous absorption resin CC using MeOH/H_2_O (0%, 30%, 50%, 80%, and 100%) to yield six fractions (F1−F6). F4 (30 g) was separated by column chromatography on silica gel, eluted with a PE–acetone gradient (20:1 to 2:1) to obtain four major fractions (Fr 4.1−4.4). Fr 4.2 (15 g) was fractionated over a silica gel column eluted with a step gradient petroleum ether–acetone solvent system (from 30:1 to 5:1) to yield three fractions (Fr B1, Fr B2, and Fr B3).

Fr B2 (9 g) was chromatographically separated over silica gel (PE–acetone, from 10:1 to 1:1) to produce three subfractions (Fr B2-1–Fr B2-3), while Fr B2-2 was separated to give three subfractions (Fr B2-2-1–Fr B2-2-3) using Sephadex LH-20 (in MeOH) columns. Fr B2-2-2 was separated using reverse-phase Semi-Prep HPLC (flow rate: 2 mL/min) under 60% C_2_H_3_N/H_2_O to provide compounds **1** (*t_R_* = 12.3 min, 7.4 mg), **2** (*t_R_* = 17.2 min, 9.0 mg), **3** (*t_R_* = 25.8 min, 10.0 mg), **4** (*t_R_* = 14.5 min, 11 mg), and **5** (*t_R_* = 13.8 min, 5.4 mg).

### 4.4. Characterization of Compounds

Pararubin W (**1**): colorless gelatinous; [*α*${]}_{D}^{21}$-101 (C 1.0. MeOH); IR (KBr) *ν_max_* 3468, 2936, 2935, 1751, 1716, 1648, and 1457 cm^−1^; UV (MeOH) λmax (log ε) 214 nm; ^1^H NMR (500 MHz, CDCl_3_) and ^13^C NMR data (125 MHz, CDCl_3_) (see Table 1 and Table 2); and HRESIMS *m*/*z* 549.2673 [M + Na]^+^ (calculated for C_27_H_42_O_10_Na, 549.2670).

Pararubin X (**2**): colorless gelatinous; [*α*${]}_{D}^{21}$-81 (C 1.0. MeOH); IR (KBr) *ν_max_* 3467, 2975, 2935, 1751, 1715, 1447, and 1457 cm^−1^; UV (MeOH) λmax (log ε) 214 nm; ^1^H NMR (500 MHz, CDCl_3_) and ^13^C NMR data (125 MHz, CDCl_3_) (see Table 1 and Table 2); and HRESIMS *m*/*z* 541.3002 [M + H]^+^ (calculated for C_28_H_45_O_10_, 541.3007).

Pararubin Y (**3**): colorless gelatinous; [*α*${]}_{D}^{21}$-87 (C 1.0. MeOH); IR (KBr) *ν_max_* 3484, 2968, 2931, 1721, 1648, 1457, and 1376 cm^−1^; UV (MeOH) λmax (log ε) 214 nm; ^1^H NMR (500 MHz, CDCl_3_) and ^13^C NMR data (125 MHz, CDCl_3_) (see Table 1 and Table 2); and HRESIMS *m*/*z* 591.2780 [M + Na]^+^ (calculated for C_29_H_44_O_11_Na, 591.2776).

Pararubin Z (**4**): colorless gelatinous; [*α*${]}_{D}^{21}$-62 (C 1.0. MeOH); IR (KBr) *ν_max_* 3737, 3433, 2963, 2930, 1718, 1647, and 1595 cm^−1^; UV (MeOH) λmax (log ε) 214 nm; ^1^H NMR (500 MHz, CDCl_3_) and ^13^C NMR data (125 MHz, CDCl_3_) (see Table 1 and Table 2); and HRESIMS *m*/*z* 549.2675 [M + Na]^+^ (calculated for C_27_H_42_O_10_Na, 549.2670).

Pararubin AA (**5**): colorless gelatinous; [*α*${]}_{D}^{21}$-86 (C 1.0. MeOH); IR (KBr) *ν_max_* 3469, 2977, 2934, 1716, 1648, 1456, and 1437 cm^−1^; UV (MeOH) λmax (log ε) 214 nm; ^1^H NMR (500 MHz, CDCl_3_) and ^13^C NMR data (125 MHz, CDCl_3_) (see Table 1 and Table 2); and HRESIMS *m*/*z* 549.2676 [M + Na]^+^ (calculated for C_27_H_42_O_10_Na, 549.2670).

### 4.5. Bacterial Culture and Anti-Chlamydial Activity Screening

*Chlamydophila abortus* is a zoonotic bacterium that mainly infects ruminants, with most cases leading to abortion. The *C. abortus* strain GN6 was previously isolated from an aborted yak fetus and propagated in the mouse embryonic fibroblast cell line McCoy [24]. Culturing of the *C. abortus* strain GN6 in McCoy as a model used for anti-chlamydial tests was described in a previous study [25]. In this study, McCoy cells were cultured in six-well plates and infected with the *C. abortus* strain GN6 at 0.5 multiplicities of infection (MOIs). Compounds **1**–**5**, with a concentration of 0−100 μg/mL, were added to the cell growth medium using DMSO (0.5%) as a vehicle. The cells were further cultured for 48 h in a 5% CO_2_ incubator at 37 °C.

The cells were then fixed with ice-cold methanol for 10 min. The chlamydial inclusions were stained with mouse anti-MOMP monoclonal antibody as the primary antibody, followed by FITC-conjugated goat anti-mouse IgG. The nuclei were stained using the fluorescent dye, 4′, 6′-diamidino-2-phenylindole (DAPI). Fluorescence images were acquired using a Leica DMI6000B fluorescence microscope. The chlamydial inclusions were counted under the fluorescence microscope, and the inclusion formation ratios (expressed as the number of inclusions/cells × 100%) were calculated in the cell cultures.

This evaluation was repeated three times to verify the anti-chlamydial effects of five compounds.

## Figures and Tables

**Table 1:** The ^1^H NMR data of compounds **1**–**5** (*δ* in ppm).

No.	1	2	3	4	5
1	5.40 dd (3.6, 3.0)	5.37 dd (3.5, 3.0)	5.43 dd (3.3, 3.0)	5.46 dd (3.4, 3.0)	5.40 dd (3.5, 2.9)
2	5.21 d (3.6)	5.11 d (3.5)	5.05 d (3.3)	5.23 d (3.4)	5.16 d (3.5)
4	4.25 dd (3.0, 3.0)	3.74 dd (3.0, 2.8)	4.96 dd (3.7, 3.2)	4.23 dd (3.0, 3.0)	4.15 dd (3.0, 2.9)
5	2.84 ddd (14.0, 13.2, 3.0), 1.77 m	2.46 ddd (13.8, 13.6, 2.8), 1.55 overlap	2.50 ddd (14.2, 13.6, 3.2), 1.67 ddd (14.2, 3.7, 3.7)	2.79 ddd (14.3, 13.5, 3.0), 1.74 ddd (14.3, 3.0, 2.8)	2.74 ddd (14.5, 13.4, 2.9), 1.66 dd (14.5, 3.0, 3.0)
6	3.19 ddd (13.2, 3.0, 3.0)	3.06 ddd (13.6, 3.0, 3.0)	2.93 ddd (13.6, 3.7, 3.0)	3.18 ddd (13.5, 3.0, 2.8)	3.13 ddd (13.4, 3.0, 2.9)
8	5.59 dd (11.0, 2.5)	5.57 dd (11.0, 2.5)	5.50 dd (10.9, 2.7)	3.97 dd (10.3, 2.8)	4.00 dd (8.9, 5.1)
9	2.08 m, 1.57 m	2.08 m, 1.57 m	2.03 m, 1.56 m	1.96 m, 1.74 m	1.94 m, 1.78 m
10	3.36 dd (10.7, 1.7)	3.46 dd (10.6, 1.7)	3.36 dd (10.6, 1.7)	5.20 dd (10.3, 1.9)	4.73 overlap
12	1.15 s	1.12 s	1.14 s	1.20 s	1.09 s
13	1.17 s	1.16 s	1.17 s	1.20 s	1.09 s
14	5.11 br s, 5.29 br s	5.09 br s, 5.24 br s	5.09 br s, 5.27 br s	5.15 br s, 4.97 br s	5.00 br s, 4.96 br s
15	1.32 s	1.21 s	1.14 s	1.31 s	1.22 s
3′	6.13 dq (7.3, 1.5)	6.08 dq (7.3, 1.6)	6.13 dd (7.2, 1.8)	6.12 dd (7.3, 1.5)	6.02 dd (7.2, 1.6)
4′	2.03 dd (7.3, 1.5)	1.94 dt (7.3, 1.8)	1.96 dd (7.2, 1.8)	1.98 dd (7.3, 1.5)	1.88 td (7.2, 1.6)
5′	1.91 t (1.5)	1.91 t (1.7)	2.02 t (1.8)	1.93 t (1.5)	1.82 t (1.6)
3″	6.13 dq (7.3, 1.5)	6.12 dd (7.3, 1.8)	6.13 dd (7.2, 1.8)	6.12 dq (7.3, 1.5)	6.04 dd (7.2, 1.6)
4″	1.98 dd (7.3, 1.5)	1.98 dt (7.3, 1.8)	1.98 dd (7.2, 1.8)	1.98 dd (7.3, 1.5)	1.88 dt (7.2, 1.6)
5″	1.86 t (1.5)	1.86 t (1.7)	1.87 t (1.8)	1.86 t (1.5)	1.77 t (1.6)
2‴	2.01 s	2.00 s	2.02 s,	2.01 s	1.94 s
2⁗			2.13 s		
OCH_3_		3.22 s			

**Table 2:** The ^13^C NMR data of compounds **1**–**5** (*δ* in ppm).

No.	1	2	3	4	5
1	71.5	72.3	71.5	71.7	72.8
2	72.3	73.5	73.7	72.5	72.5
3	74.4	74.3	72.7	74.5	74.5
4	66.6	75.1	77.3	66.6	66.8
5	31.4	31.4	27.6	30.9	31.4
6	36.4	35.5	36.8	36.4	34.5
7	148.2	148.9	148.8	151.6	149.4
8	75.8	76.2	75.8	71.6	74.9
9	37.2	37.2	37.1	37.2	36.4
10	75.4	73.7	75.5	77.6	77.9
11	73.4	78.2	73.4	72.7	72.7
12	25.6	21.0	25.8	25.7	26.4
13	25.1	21.4	24.9	26.5	25.8
14	115.8	115.3	115.5	112.8	115.9
15	25.0	23.6	23.3	25.0	25.0
1′	168.7	168.6	168.8	169.4	169.1
2′	129.1	129.2	129.2	129.4	129.4
3′	139.7	139.4	139.7	139.8	138.8
4′	16.2	16.2	16.2	16.1	16.0
5′	20.9	20.6	20.9	21.0	20.9
1″	168.1	168.3	168.2	168.2	168.2
2″	128.8	129.1	128.9	128.8	128.8
3″	139.5	139.0	139.5	138.9	139.9
4″	16.0	16.0	16.1	16.0	16.0
5″	20.6	20.5	20.6	20.6	20.7
1‴	171.8	171.8	171.6	172.0	172.3
2‴	21.0	21.0	21.1	21.0	21.1
1⁗			172.0		
2⁗			21.1		
OCH_3_		49.6			

## Data Availability

Data are contained within the article and Supplementary Materials.

## Supplementary Materials

The following supporting information can be downloaded at https://www.mdpi.com/article/10.3390/ijms26031388/s1.

## Abbreviations

DAPI	4′, 6′-diamidino-2-phenylindole
DMSO	Dimethyl sulfoxide
FITC	Fluorescein isothiocyanate
HRESIMS	High-resolution electrospray ionization mass spectrometry
IR	Infrared spectroscopy
MCI	Middle chromatogram isolated
MOI	Multiplicities of infection
MOMP	Major outer-membrane protein
NMR	Nuclear magnetic resonance spectroscopy
UV	Ultraviolet
TLC	Thin-layer chromatography
TMS	Tetramethyl silane
2D	Two-dimensional
1D	One-dimensional
