# Supplementary material for: Bisabolane Sesquiterpenes with Anti-Chlamydial Activity Isolated from Ligularia narynensis
Source: Int J Mol Sci. 2025 Feb 6;26(3):1388. doi: 10.3390/ijms26031388 (PMC11818376; doi:10.3390/ijms26031388)
Supplement: Supplementary file 1 [file ijms-26-01388-s001.zip › ijms-3436484-supplementary.pdf]

List of Supplementary Material

Figure S1. ^{1}H-NMR spectrum of **1** in MeOD (500 MHz)....................S2
Figure S2. ^{13}C-NMR spectrum of **1** in MeOD (125 MHz)....................S2
Figure S3. ^{1}H-^{1}H COSY spectrum of **1** in MeOD (500 MHz)....................S3
Figure S4. HSQC spectrum of **1** in MeOD (125 MHz)....................S3
Figure S5. HMBC spectrum of **1** in MeOD (125 MHz)....................S4
Figure S6. NOESY spectrum of **1** in MeOD (500 MHz)....................S4
Figure S7. HR-ESI-MS spectrum of **1**....................S5
Figure S8. IR spectrum of **1**....................S5
Figure S9. ^{1}H-NMR spectrum of **2** in MeOD (500 MHz)....................S6
Figure S10. ^{13}C-NMR spectrum of **2** in MeOD (125 MHz)....................S6
Figure S11. ^{1}H-^{1}H COSY spectrum of **2** in MeOD (500 MHz)....................S7
Figure S12. HSQC spectrum of **2** in MeOD (125 MHz)....................S7
Figure S13. HMBC spectrum of **2** in MeOD (125 MHz)....................S8
Figure S14. NOESY spectrum of **2** in MeOD (500 MHz)....................S8
Figure S15. HR-ESI-MS spectrum of **2**....................S9
Figure S16. IR spectrum of **2**....................S9
Figure S17. ^{1}H-NMR spectrum of **3** in MeOD (500 MHz)....................S10
Figure S18. ^{13}C-NMR spectrum of **3** in MeOD (125 MHz)....................S10
Figure S19. ^{1}H-^{1}H COSY spectrum of **3** in MeOD (500 MHz)....................S11
Figure S20. HSQC spectrum of **3** in MeOD (125 MHz)....................S11
Figure S21. HMBC spectrum of **3** in **MeOD** (125 MHz)....................S12
Figure S22. NOESY spectrum of **3** in MeOD (500 MHz)....................S12
Figure S23. HR-ESI-MS spectrum of **3**....................S13
Figure S24. IR spectrum of **3**....................S13
Figure S25. ^{1}H-NMR spectrum of **4** in MeOD (500 MHz)....................S14
Figure S26. ^{13}C-NMR spectrum of **4** in MeOD (125 MHz)....................S14
Figure S27. ^{1}H-^{1}H COSY spectrum of **4** in **MeOD** (500 MHz)....................S15
Figure S28. HSQC spectrum of **4** in MeOD (125 MHz)....................S15
Figure S29. HMBC spectrum of **4** in MeOD (125 MHz)....................S16
Figure S30. NOESY spectrum of **4** in MeOD (500 MHz)....................S16
Figure S31. HR-ESI-MS spectrum of **4**....................S17
Figure S32. IR spectrum of **4**....................S17
Figure S33. ^{1}H-NMR spectrum of **5** in MeOD (500 MHz)....................S18
Figure S34. ^{13}C-NMR spectrum of **5** in MeOD (125 MHz)....................S18
Figure S35. ^{1}H-^{1}H COSY spectrum of **5** in MeOD (500 MHz)....................S19
Figure S36. HSQC spectrum of **5** in MeOD (125 MHz)....................S19
Figure S37. HMBC spectrum of **5** in MeOD (125 MHz)....................S20
Figure S38. NOESY spectrum of **5** in MeOD (500 MHz)....................S20
Figure S39. HR-ESI-MS spectrum of **5**....................S21
Figure S40. IR spectrum of **5**....................S21
Figure S41. The ECD spectra of compounds **1-5**....................S22

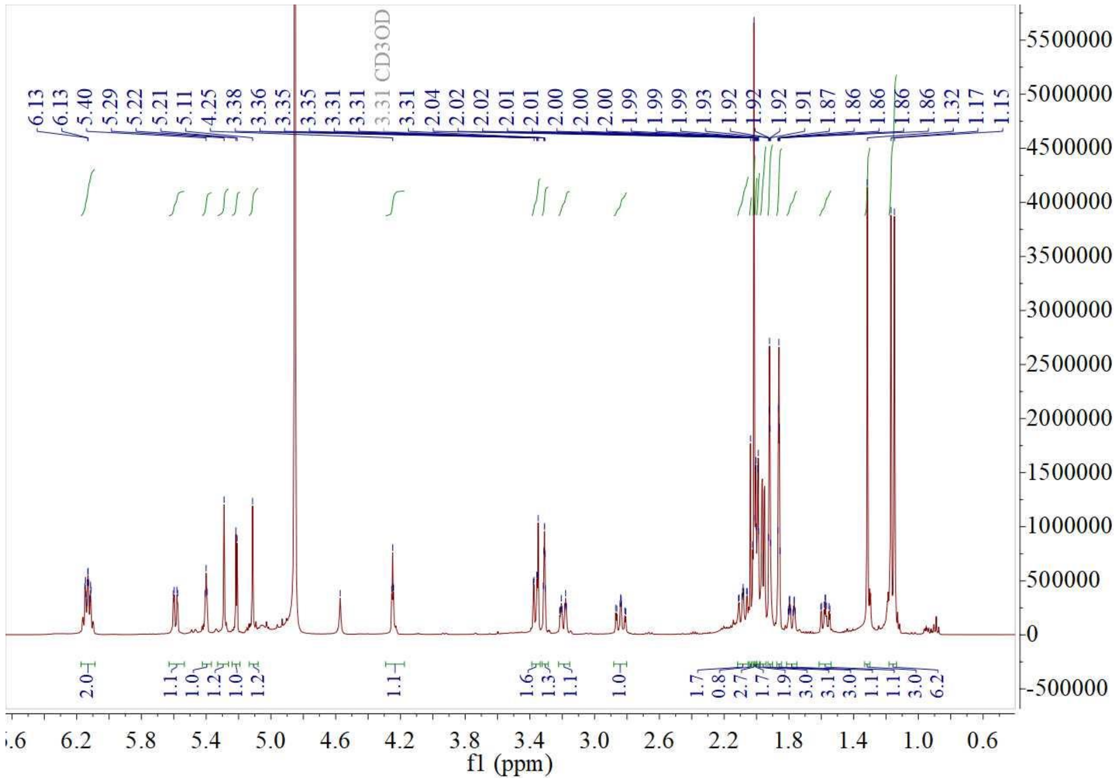

Figure S1. ^{1}H-NMR spectrum of **1** in MeOD (500 MHz)**.**

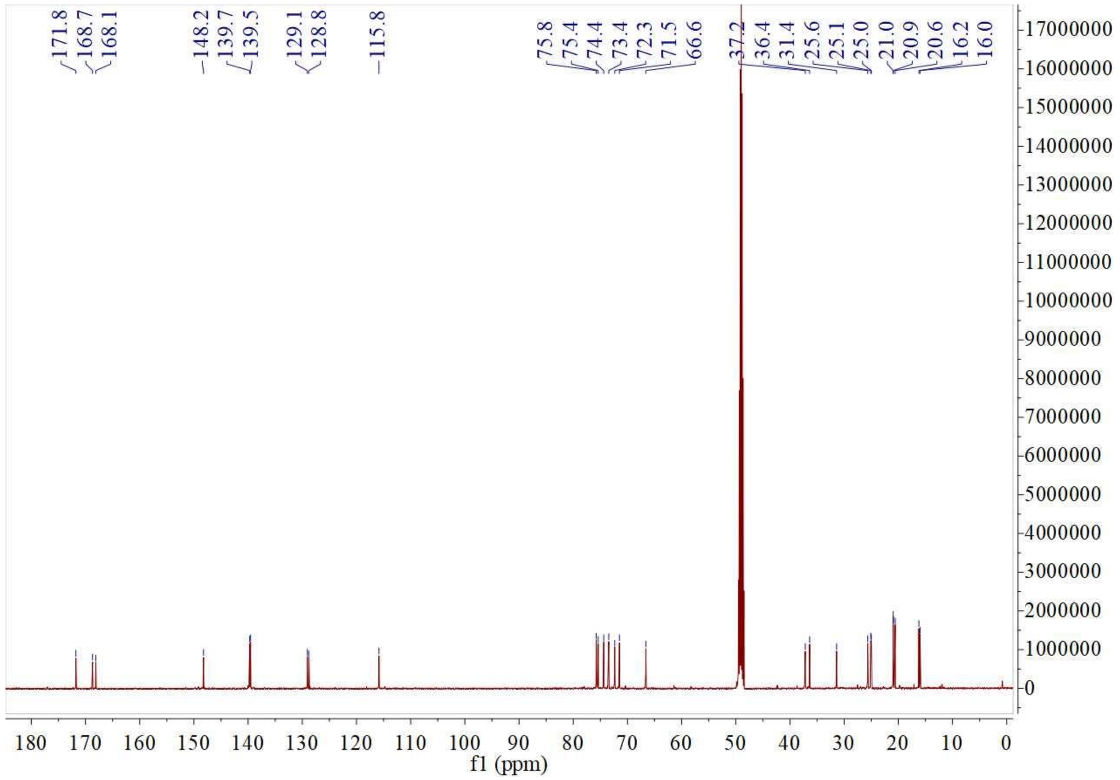

Figure S2. ^{13}C-NMR spectrum of **1** in MeOD (125 MHz)

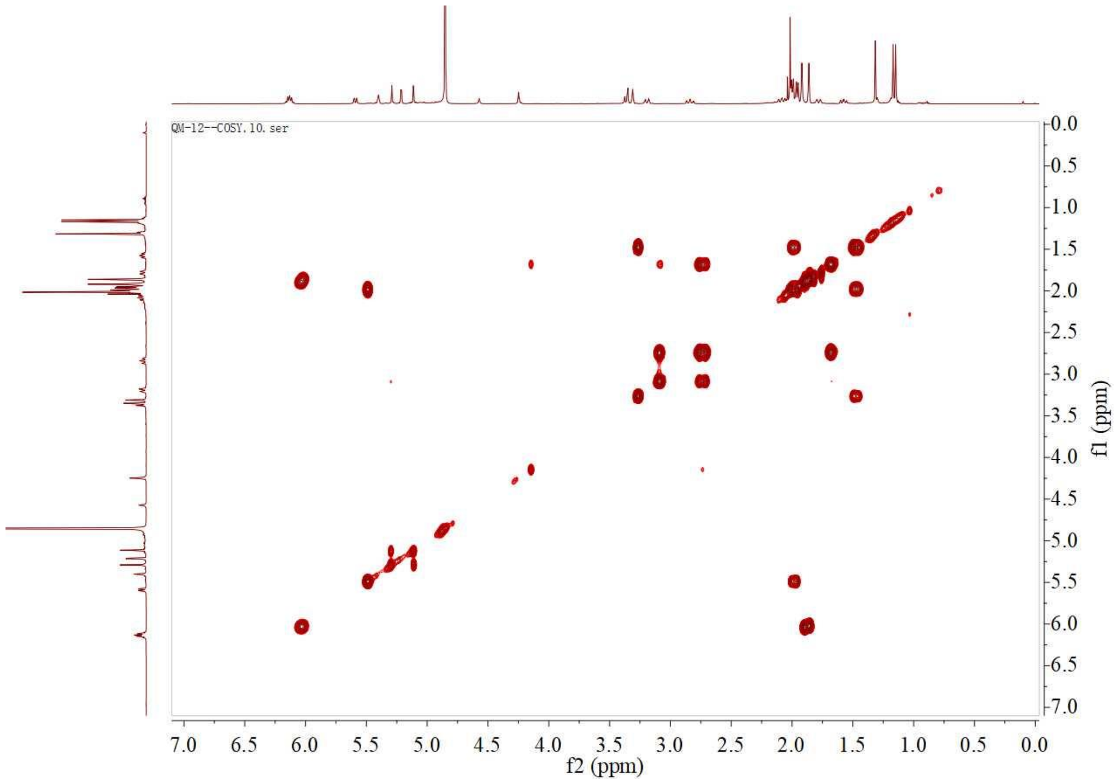

Figure S3. ^{1}H-^{1}H COSY spectrum of **1** in MeOD (500 MHz).

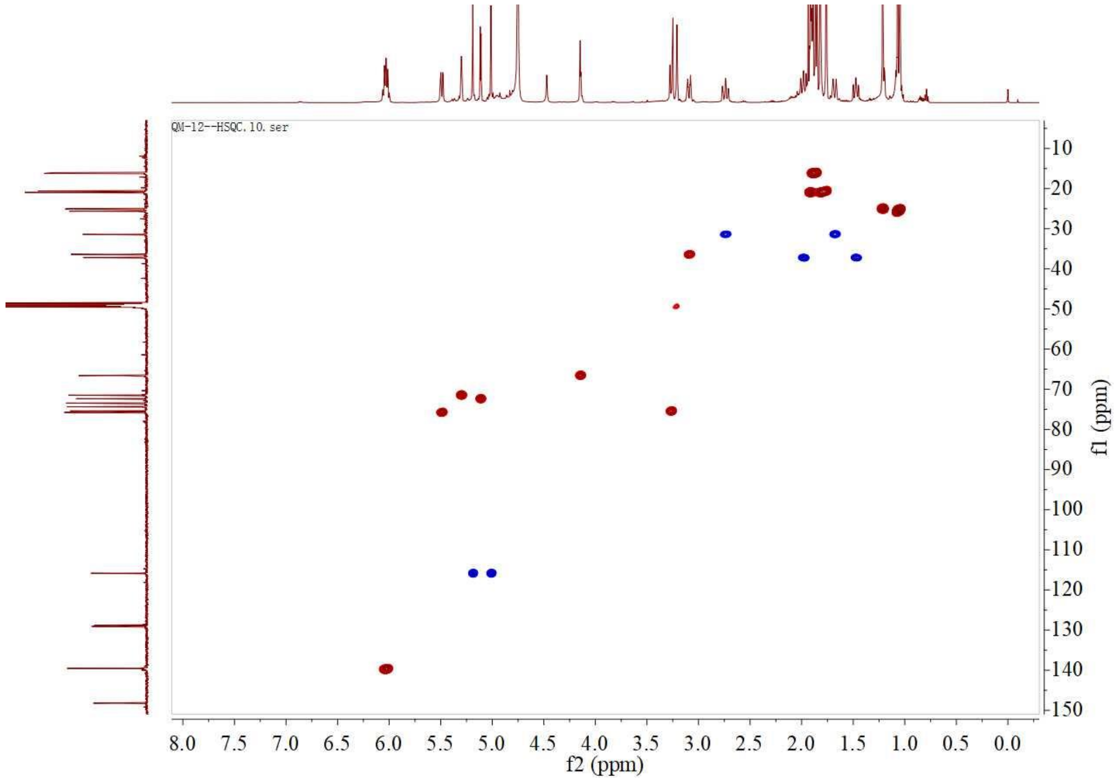

Figure S4. HSQC spectrum of **1** in MeOD (125 MHz)

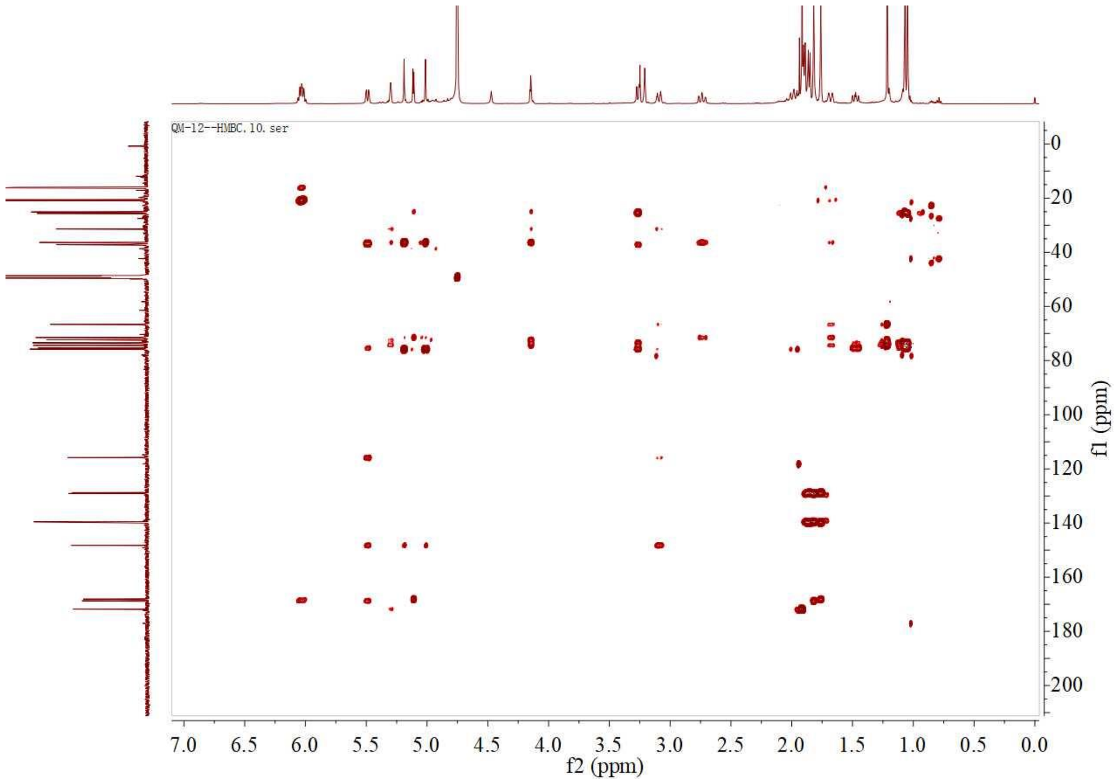

Figure S5. HMBC spectrum of **1** in MeOD (125 MHz)

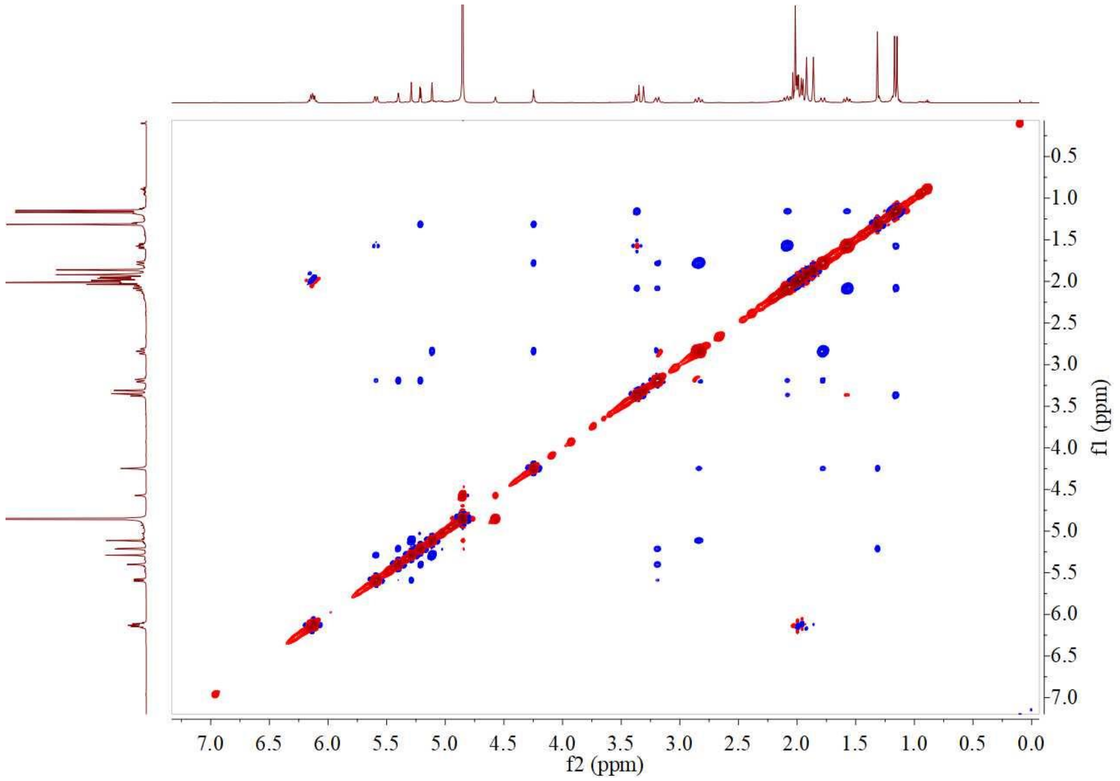

Figure S6. NOESY spectrum of **1** in MeOD (500 MHz).

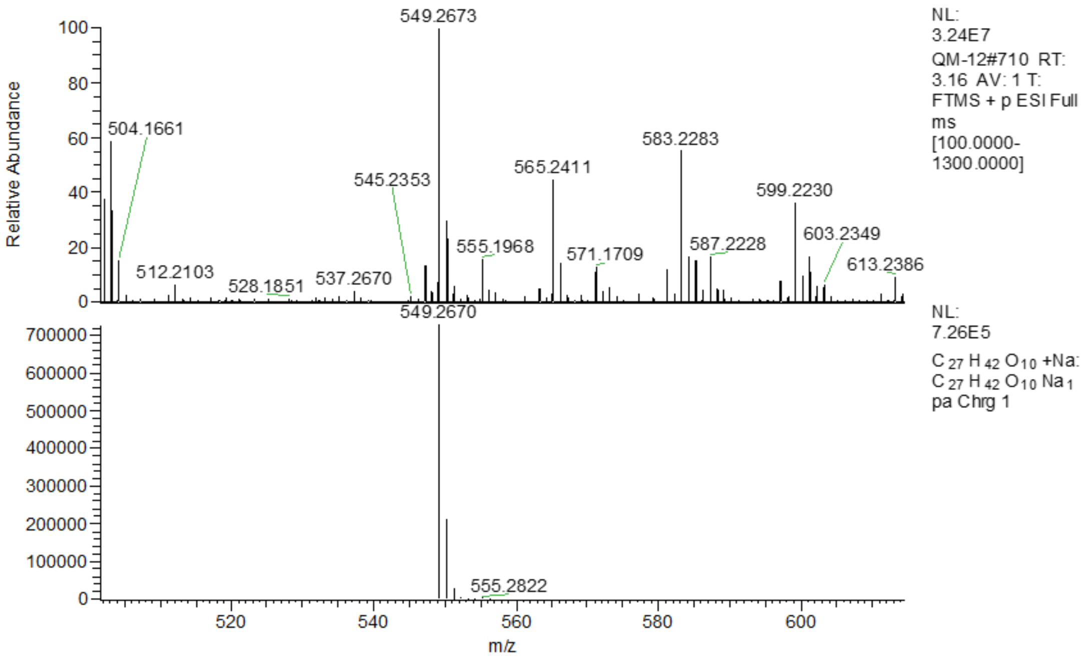

Figure S7. HR-ESI-MS spectrum of **1**

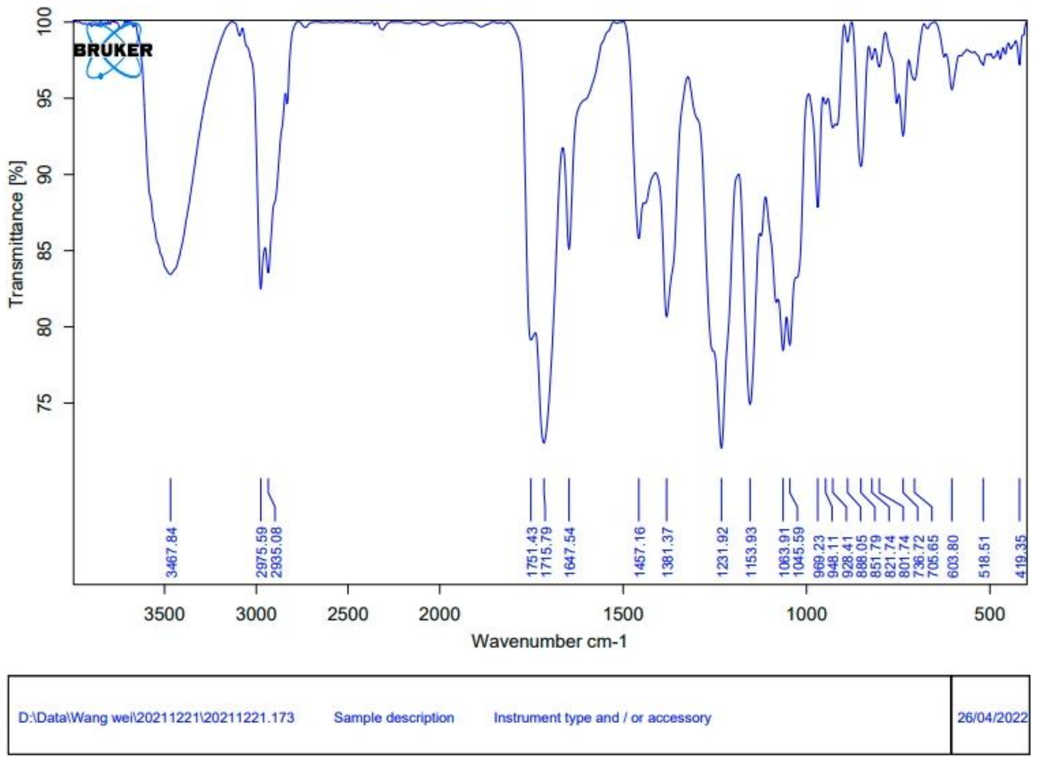

Figure S8. IR spectrum of **1**

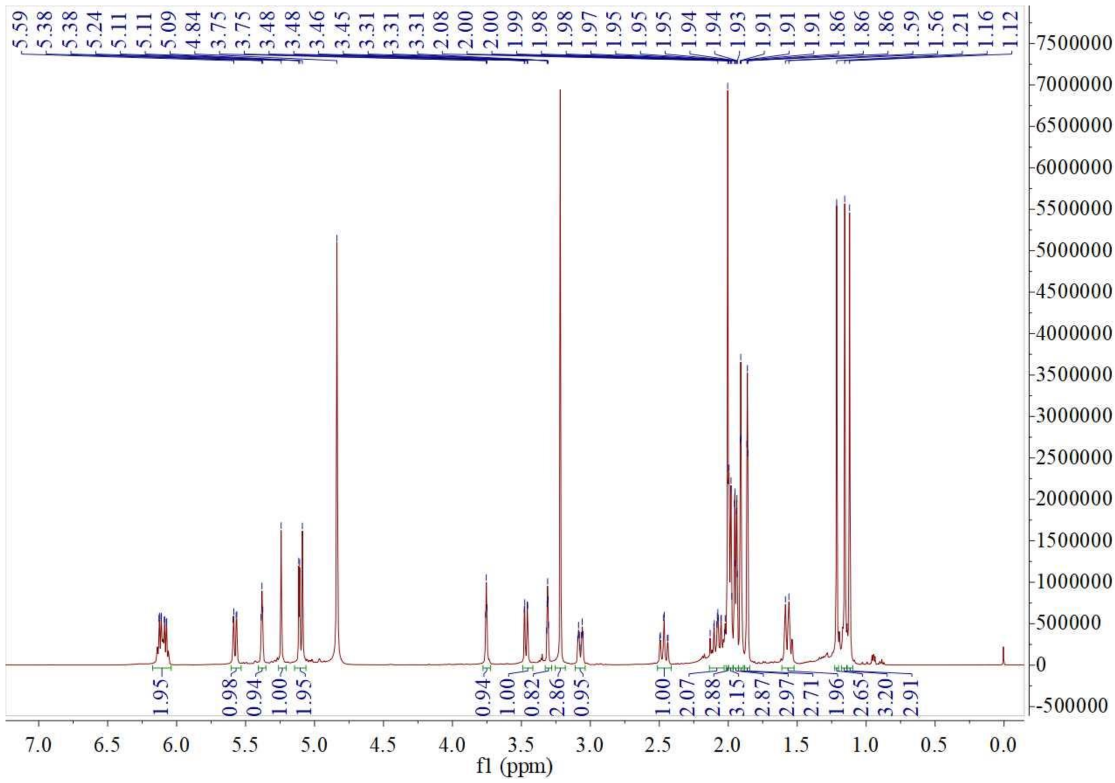

Figure S9. ^{1}H-NMR spectrum of **2** in MeOD (500 MHz).

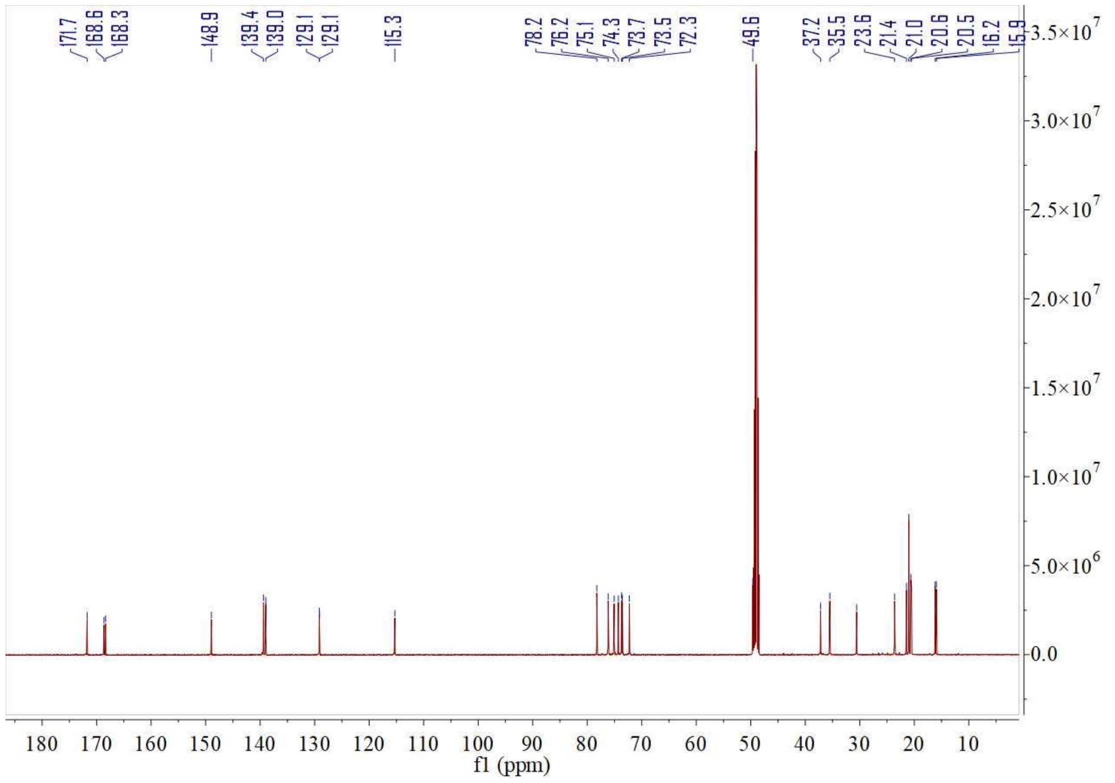

Figure S10. ^{13}C-NMR spectrum of **2** in MeOD (125 MHz)

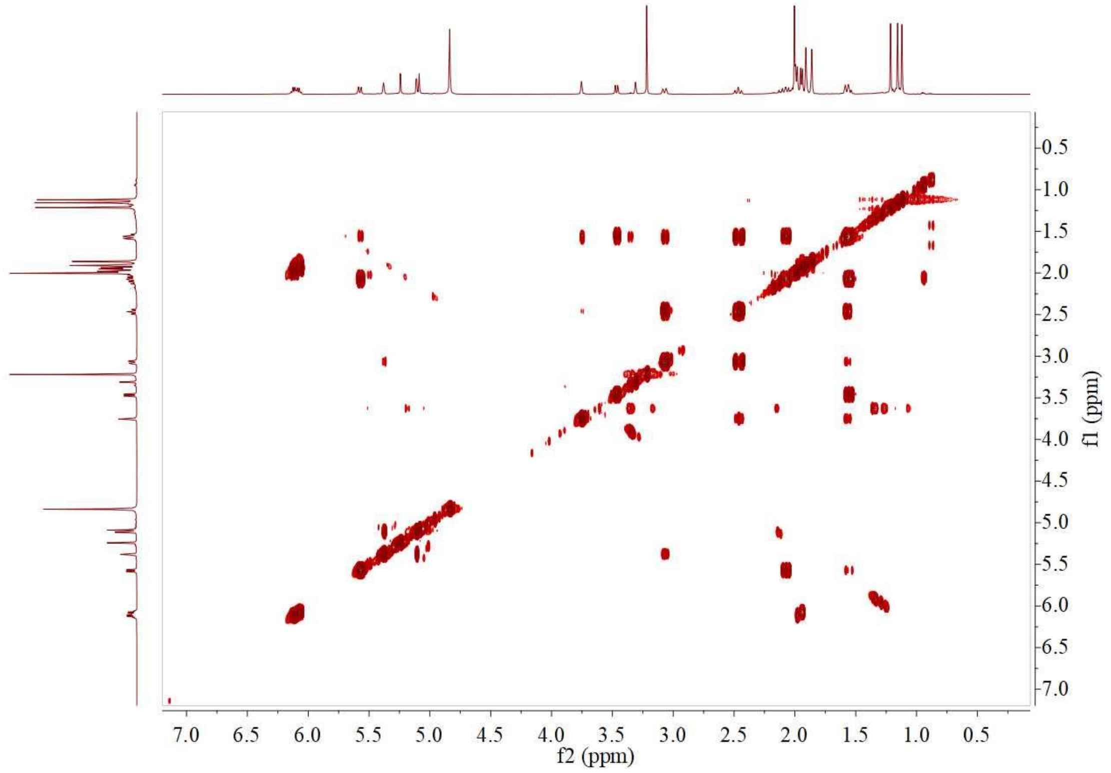

Figure S11. ^{1}H-^{1}H COSY spectrum of **2** in MeOD (500 MHz)

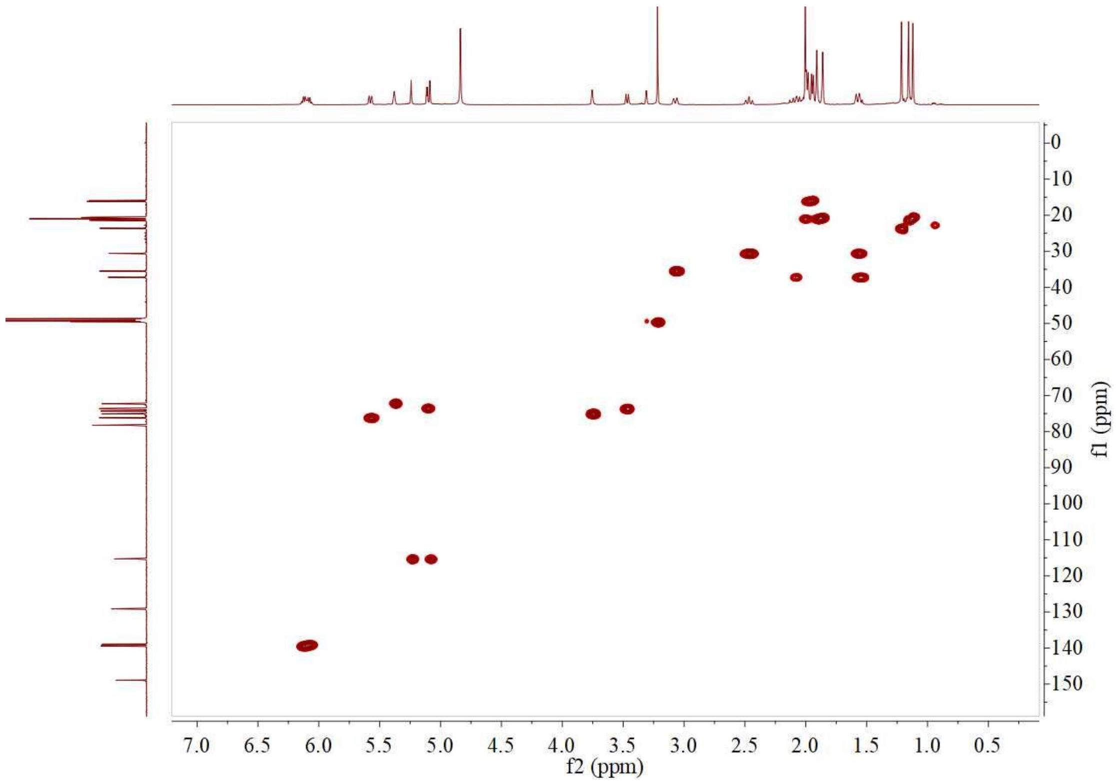

Figure S12. HSQC spectrum of **2** in MeOD (125 MHz).

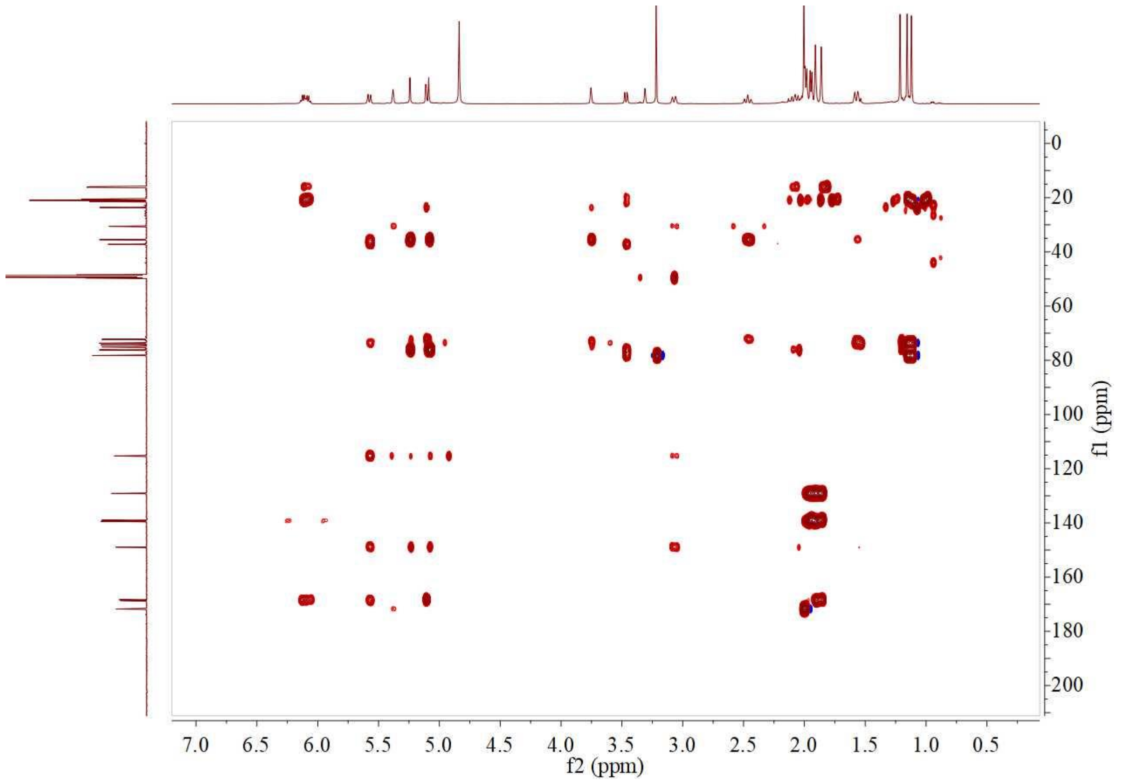

Figure S13. HMBC spectrum of **2** in MeOD (125 MHz).

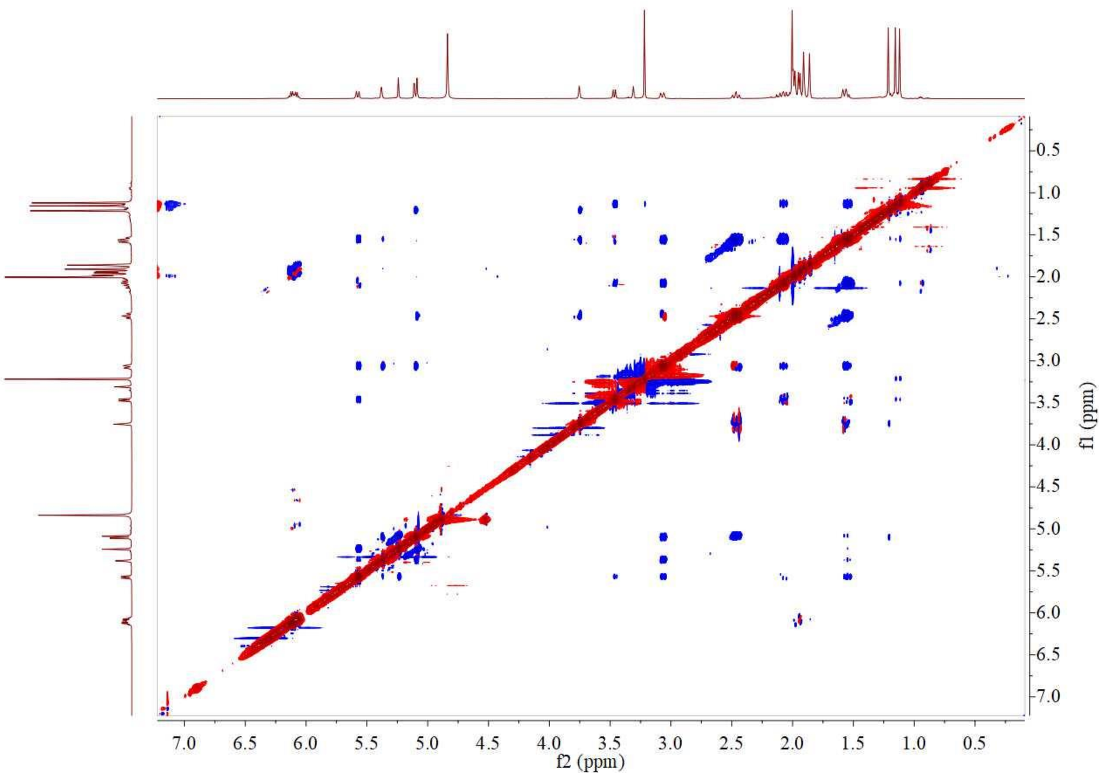

Figure S14. NOESY spectrum of **2** in MeOD (500 MHz).

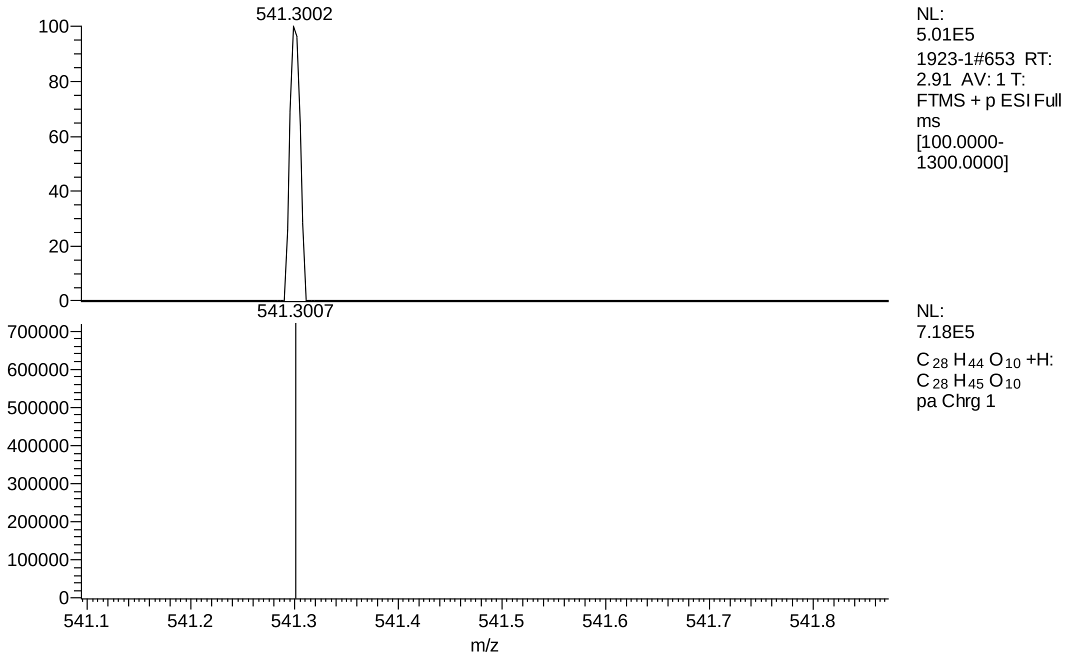

Figure S15. HR-ESI-MS spectrum of **2**

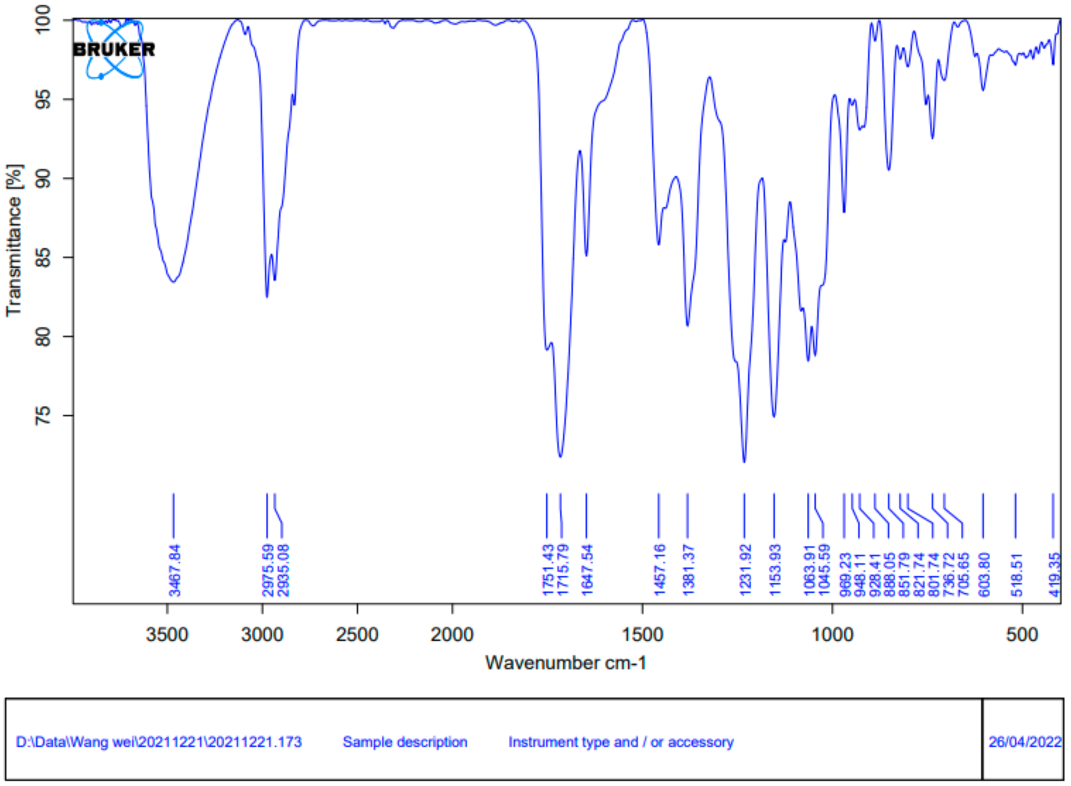

Figure S16. IR spectrum of **2**

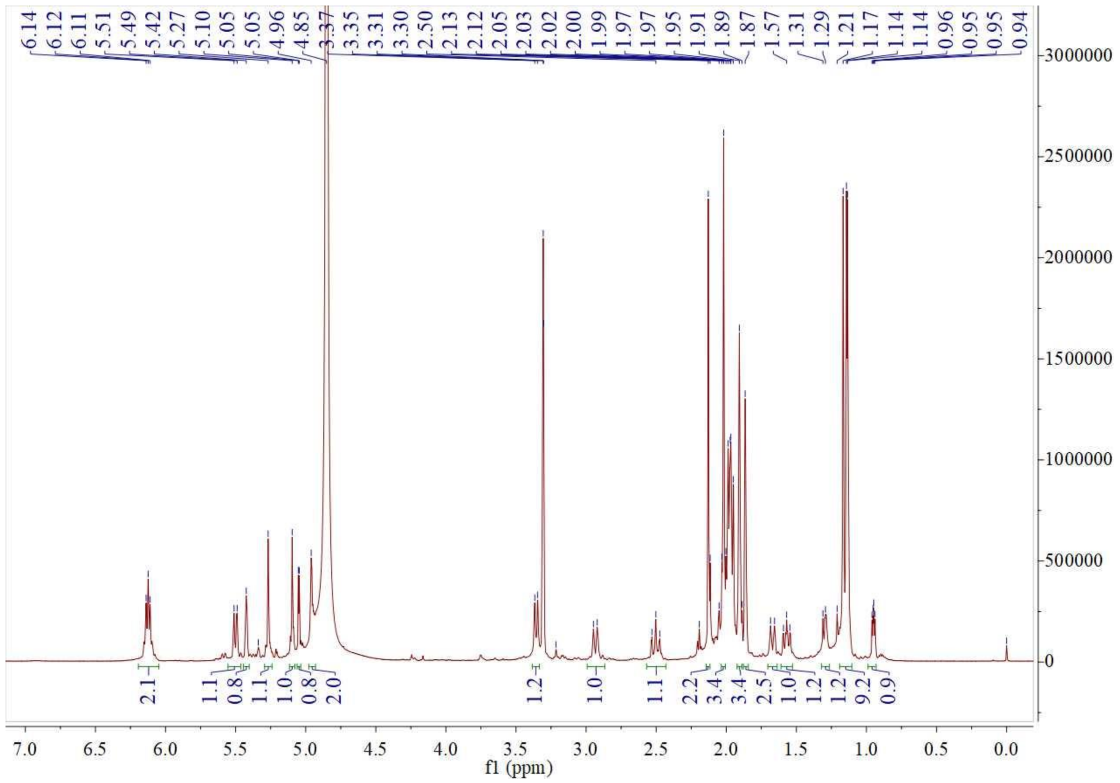

Figure S17. ^{1}H-NMR spectrum of **3** in MeOD (500 MHz)**.**

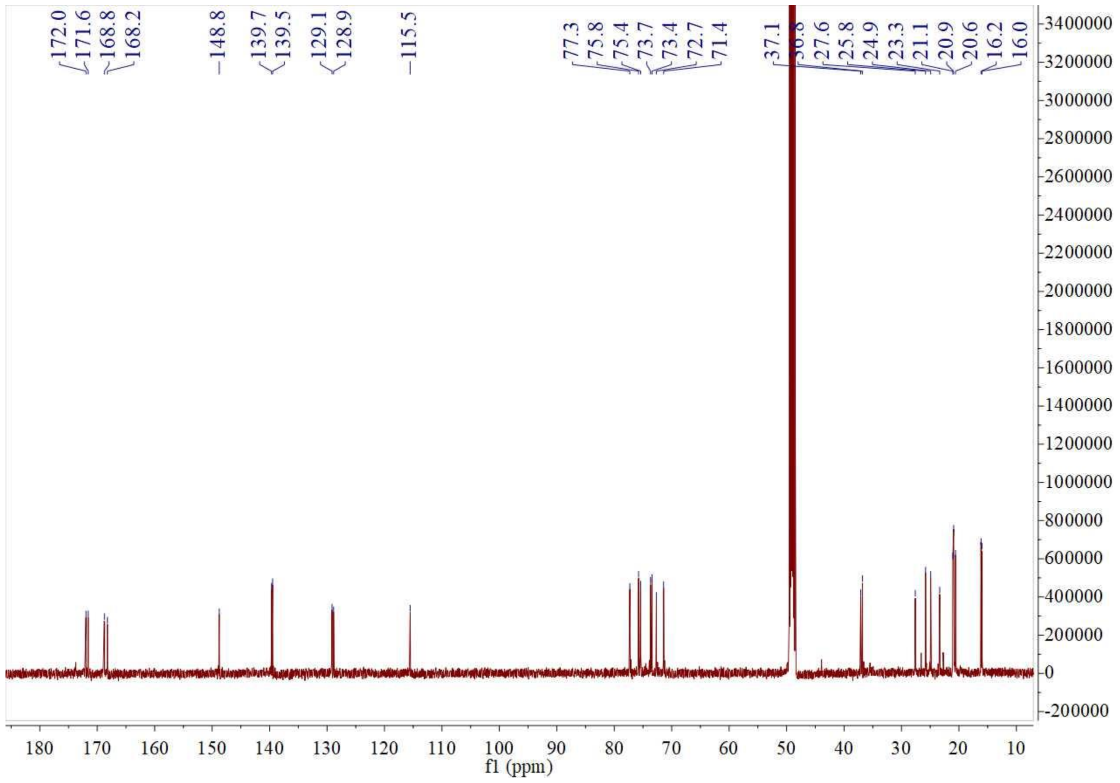

Figure S18. ^{13}C-NMR spectrum of **3** in MeOD (125 MHz)

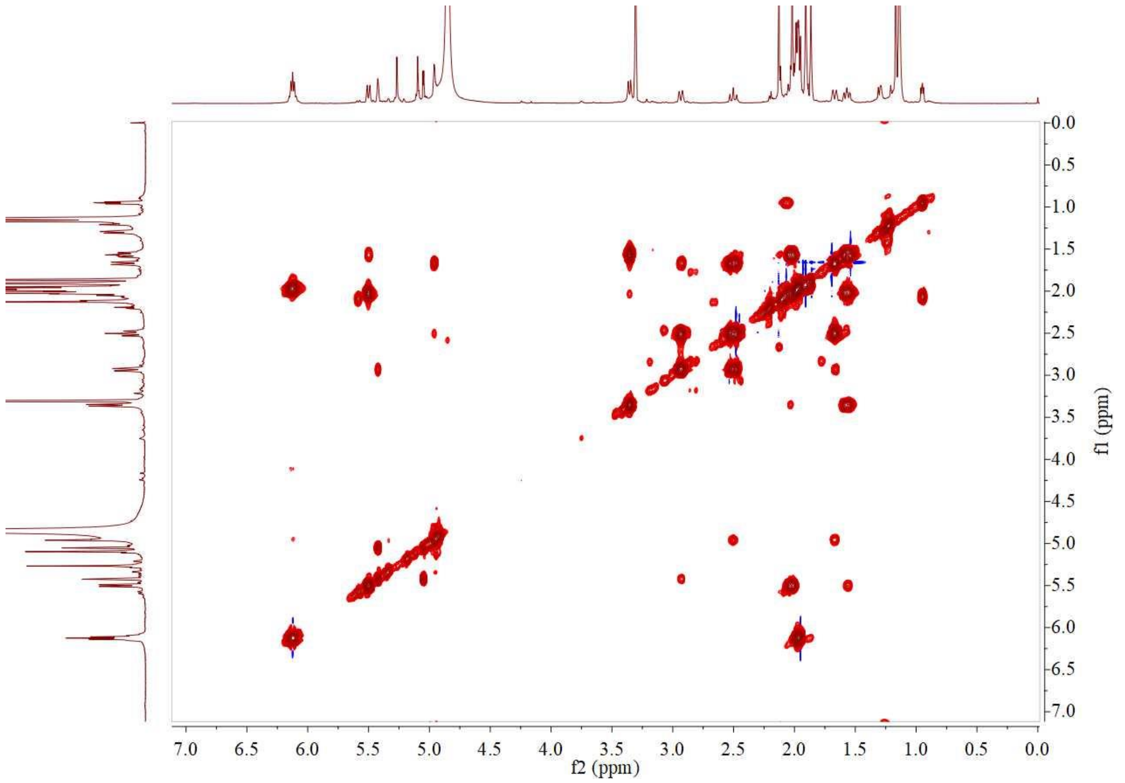

Figure S19. ^{1}H-^{1}H COSY spectrum of **3** in MeOD (500 MHz).

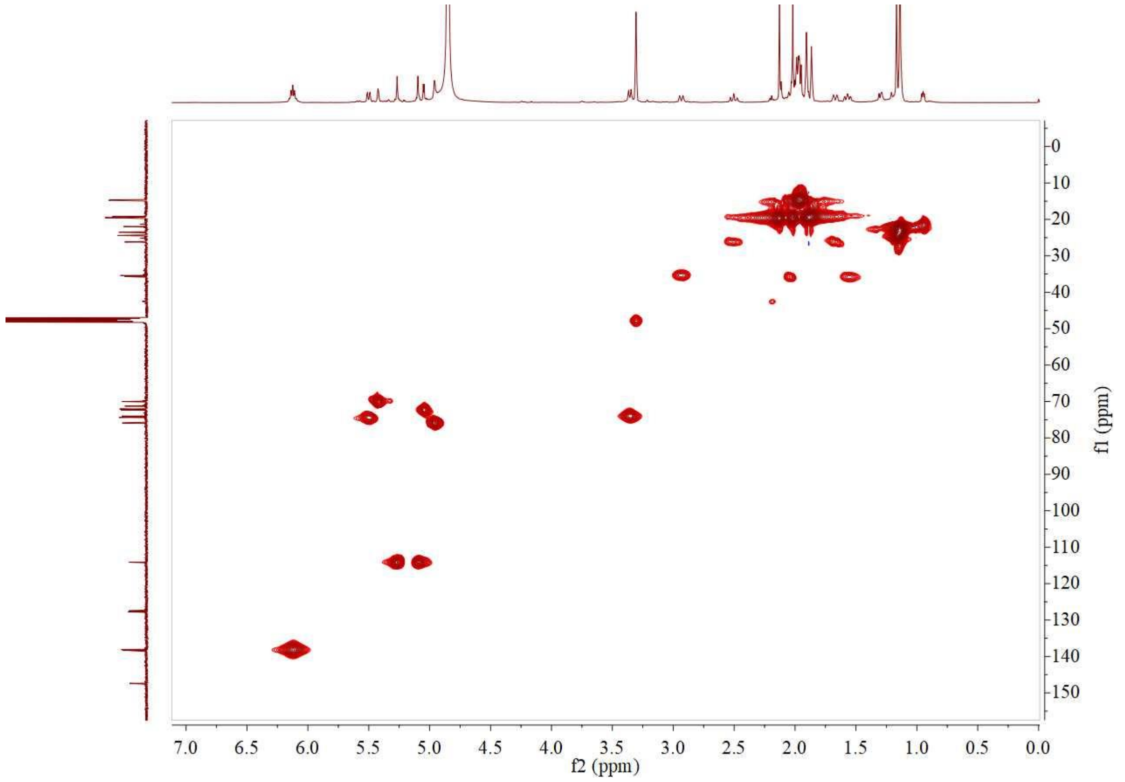

Figure S20. HSQC spectrum of **3** in MeOD (125 MHz)

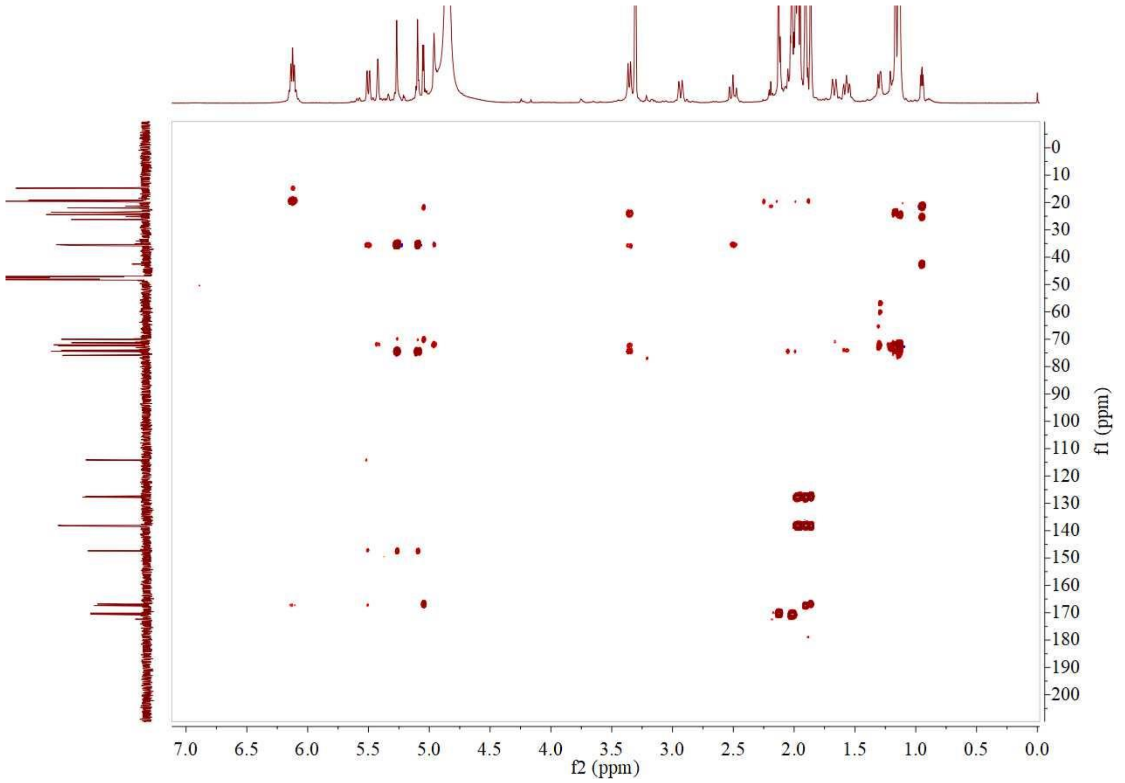

Figure S21. HMBC spectrum of **3** in **MeOD** (125 MHz)

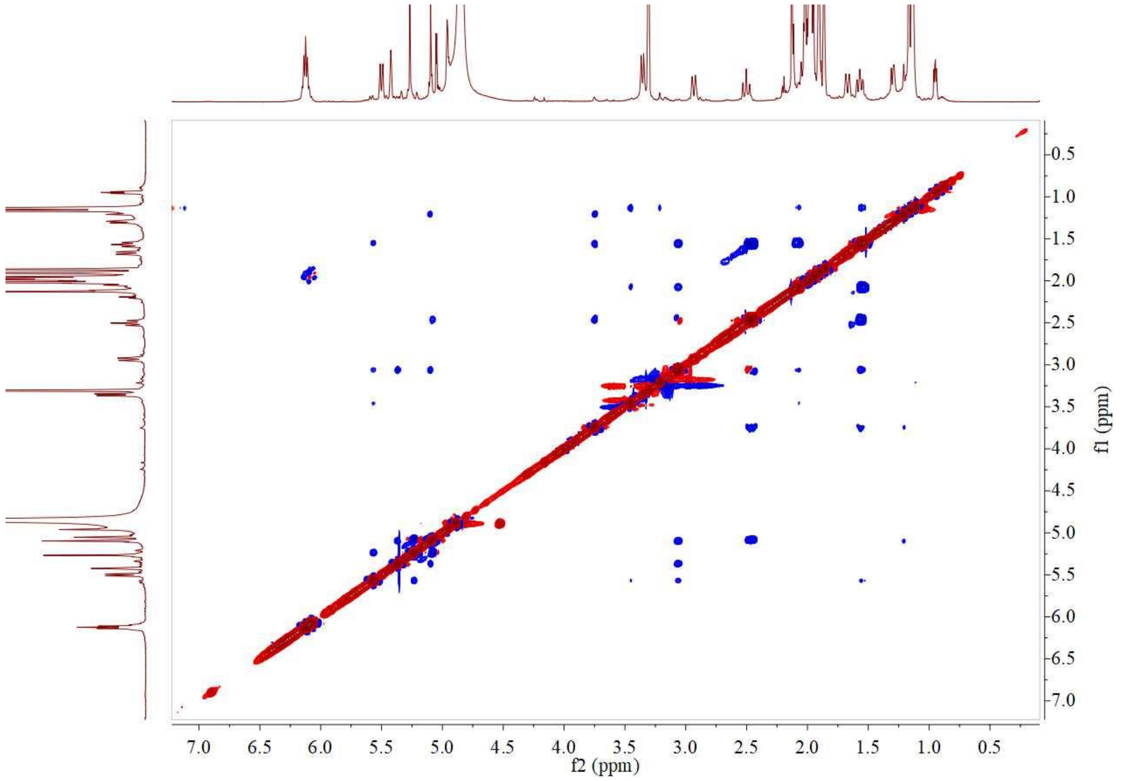

Figure S22. NOESY spectrum of **3** in MeOD (500 MHz).

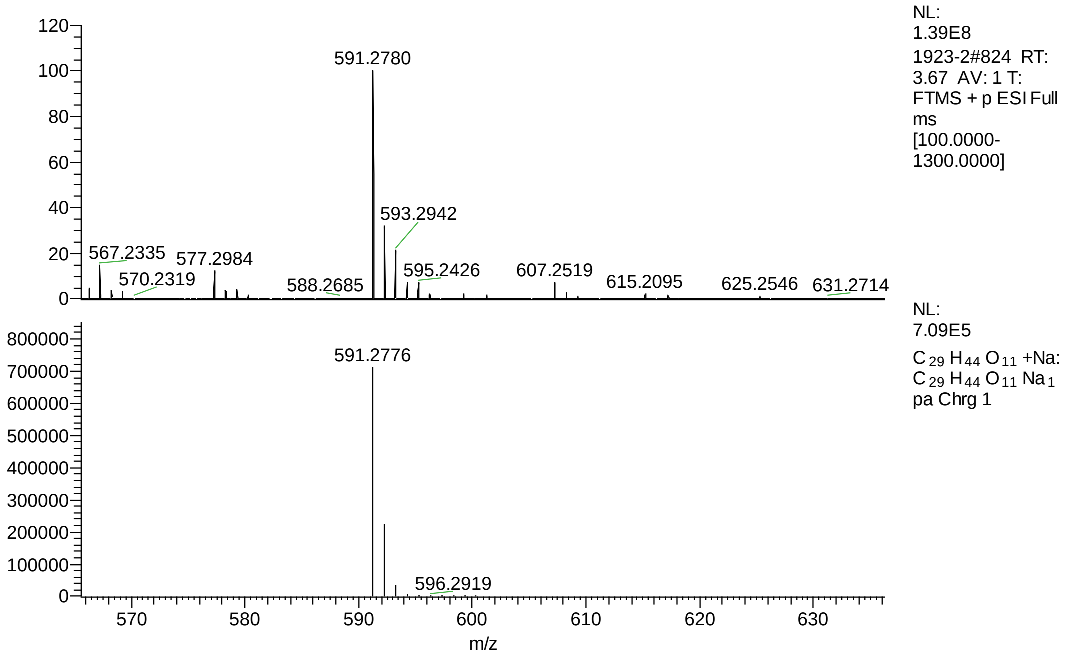

Figure S23. HR-ESI-MS spectrum of **3**

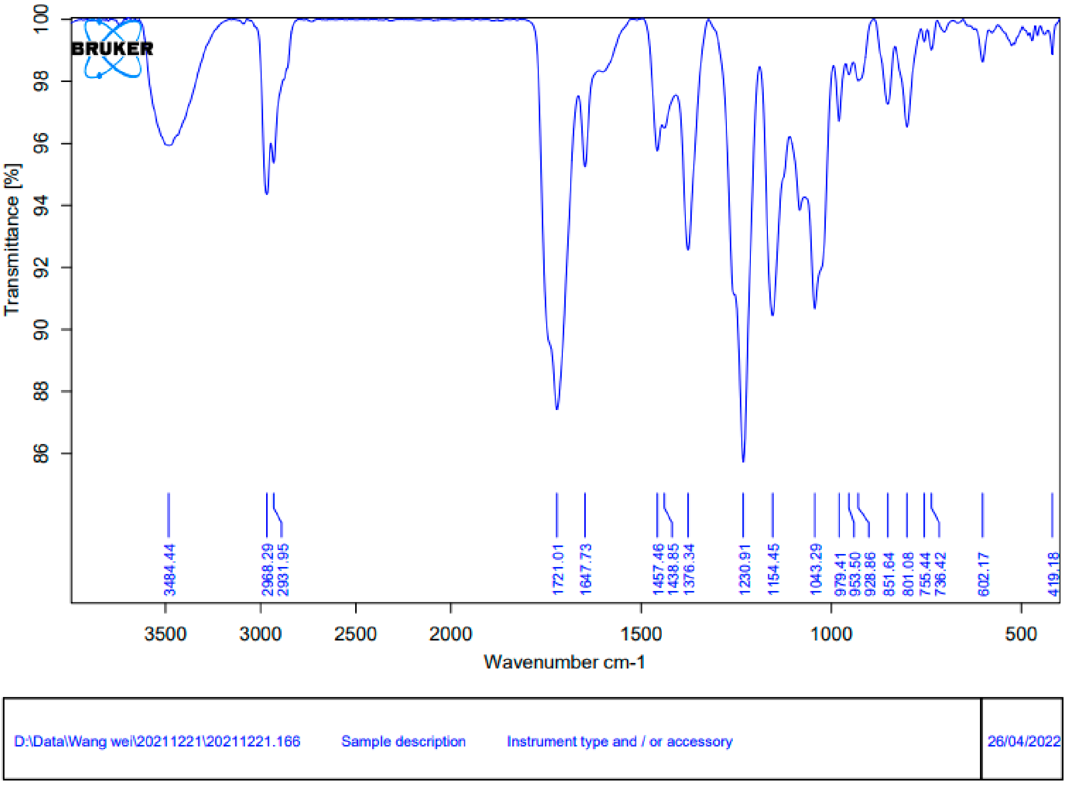

Figure S24. IR spectrum of **3**

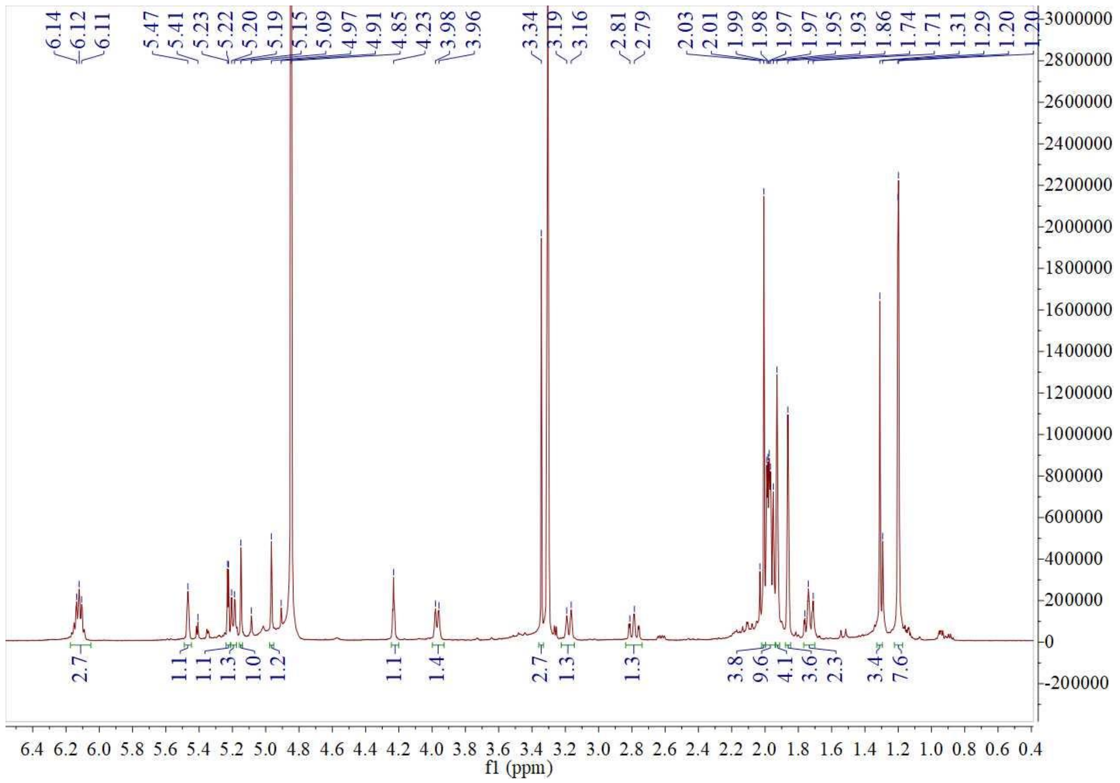

Figure S25. ^{1}H-NMR spectrum of **4** in MeOD (500 MHz).

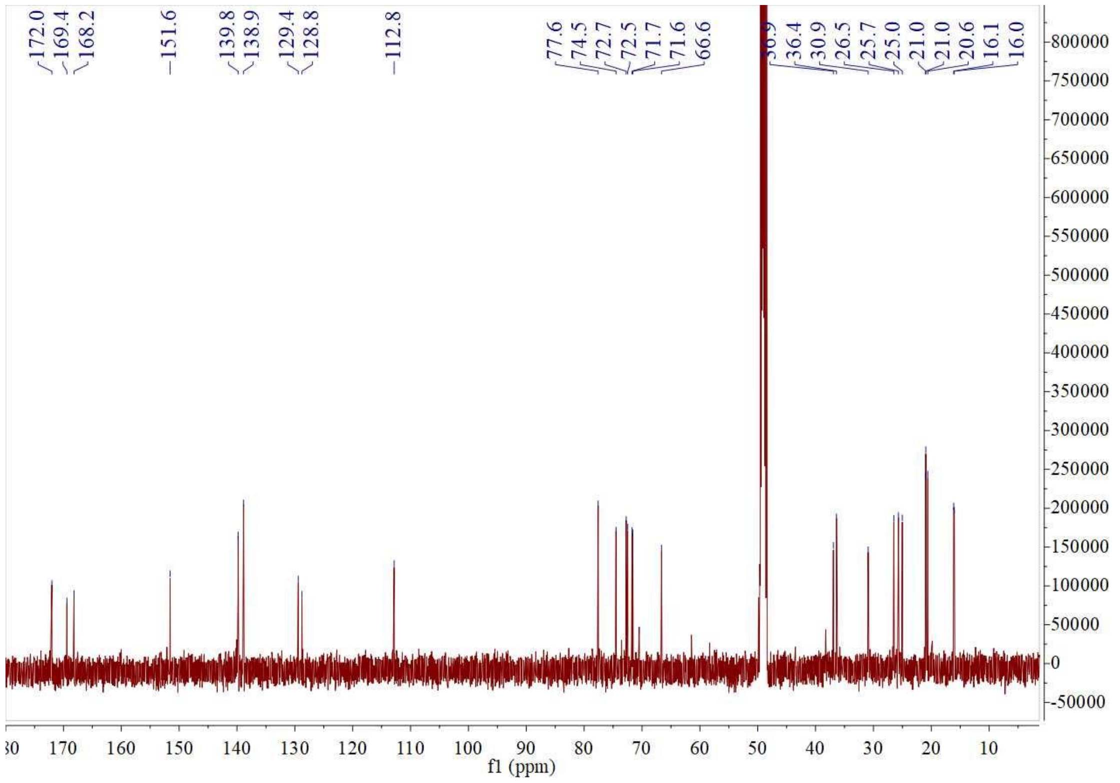

Figure S26. ^{13}C-NMR spectrum of **4** in MeOD (125 MHz)

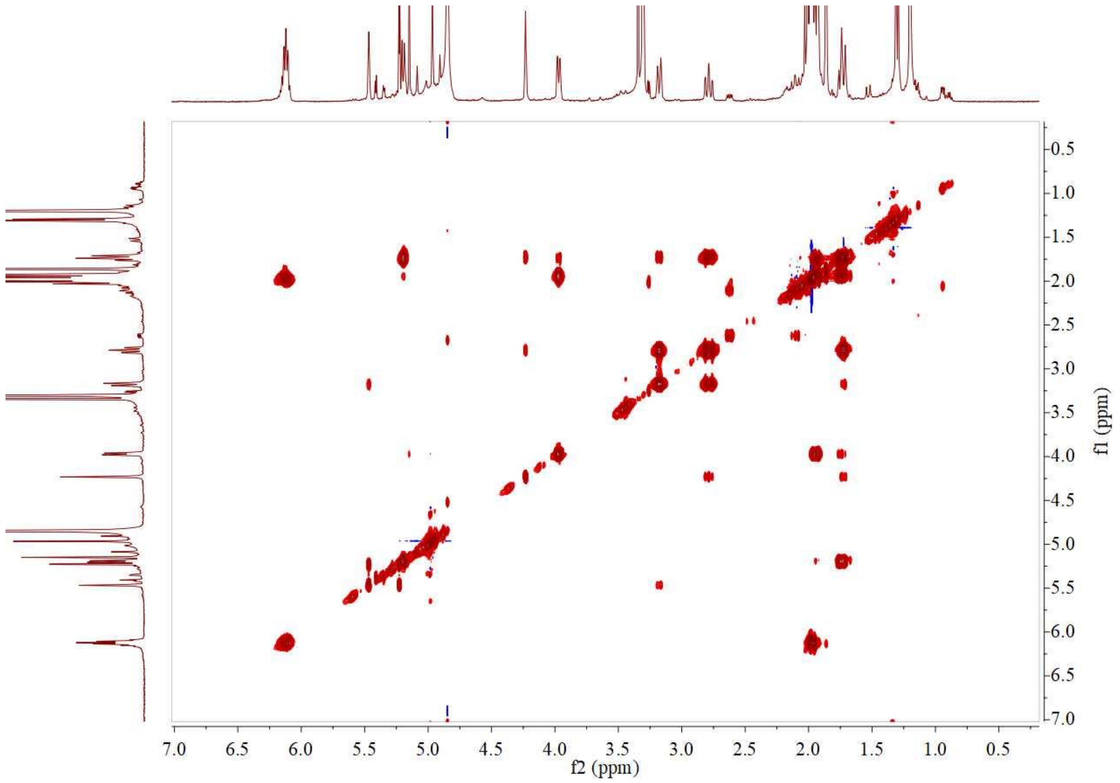

Figure S27. ^{1}H-^{1}H COSY spectrum of **4** in **MeOD** (500 MHz)

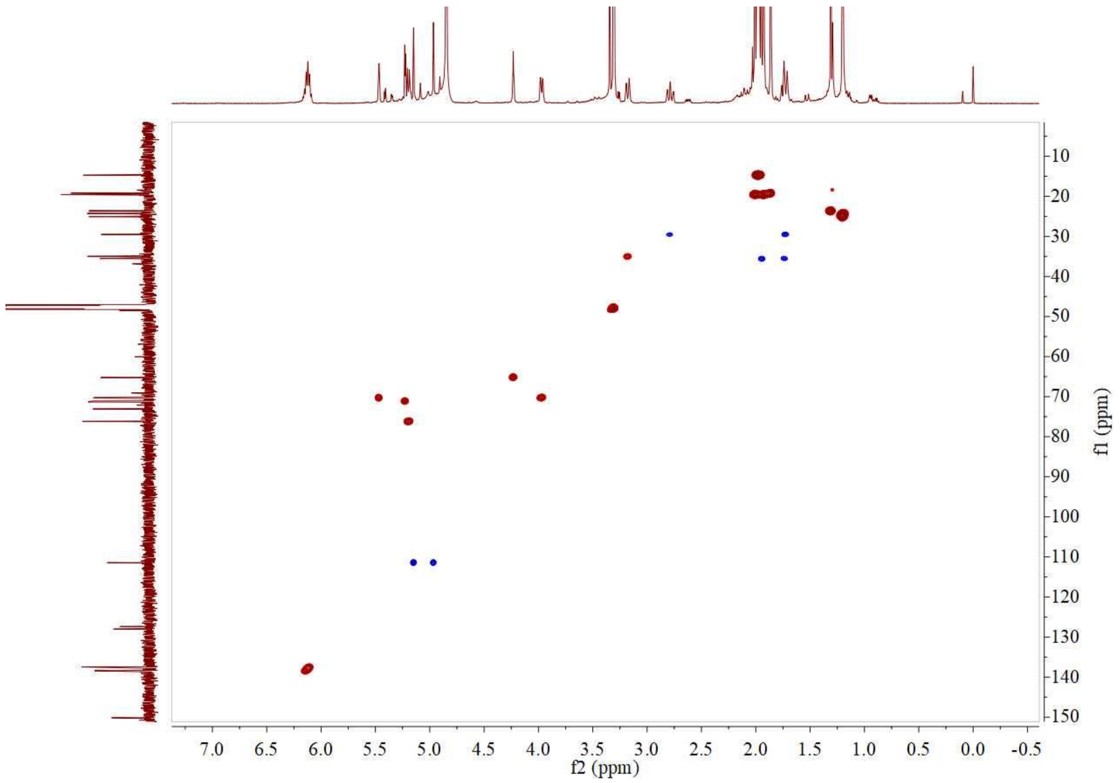

Figure S28. HSQC spectrum of **4** in MeOD (125 MHz).

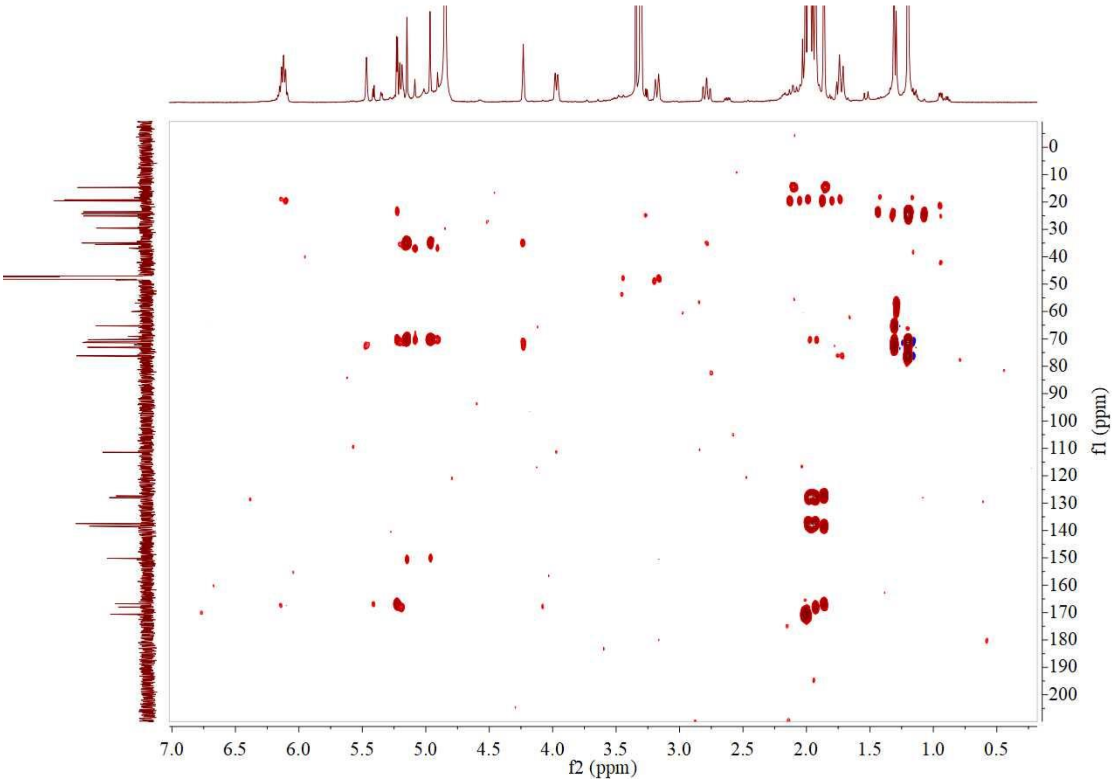

Figure S29. HMBC spectrum of **4** in MeOD (125 MHz).

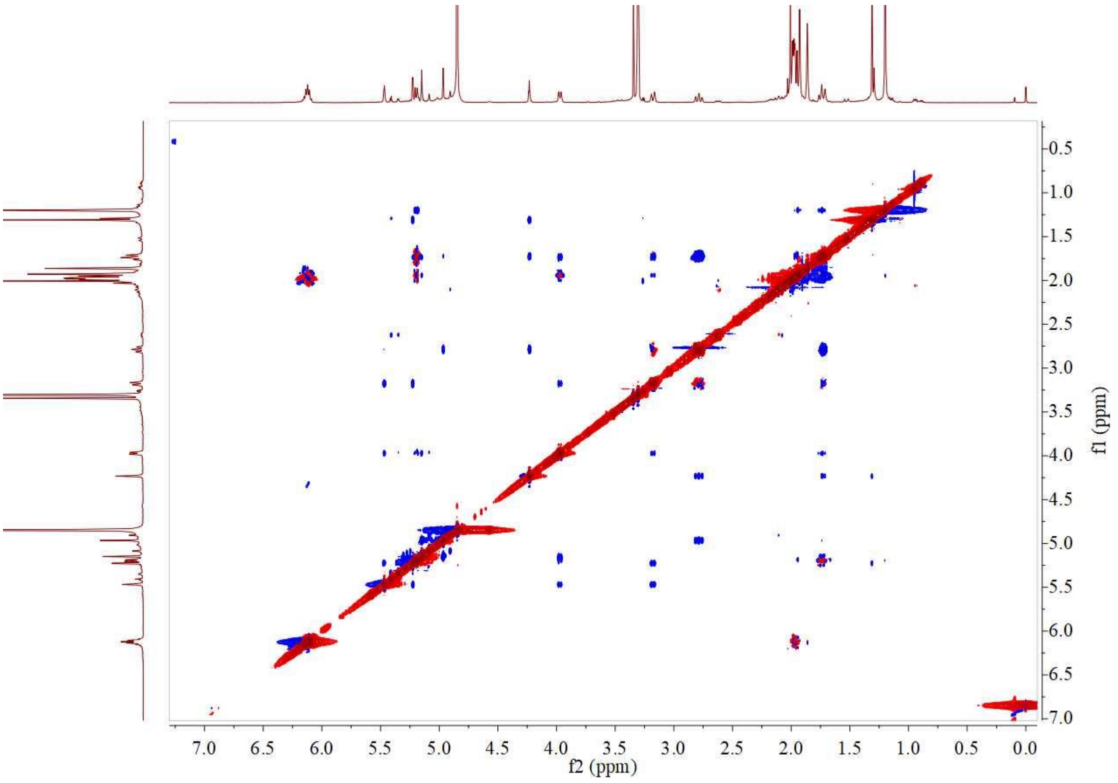

Figure S30. NOESY spectrum of **4** in MeOD (500 MHz).

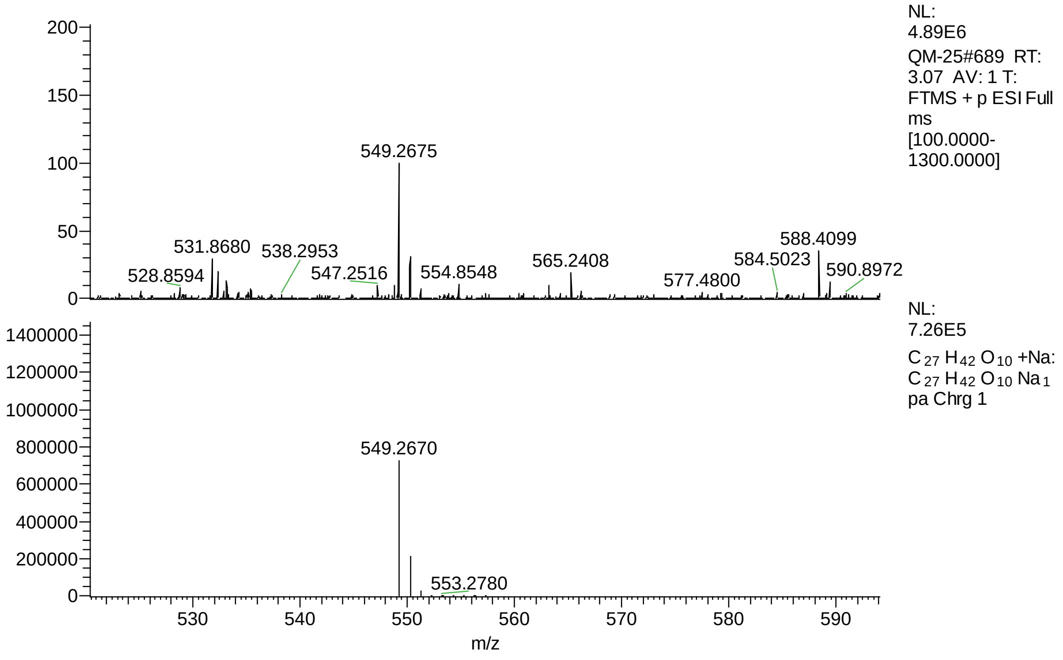

Figure S31. HR-ESI-MS spectrum of **4**

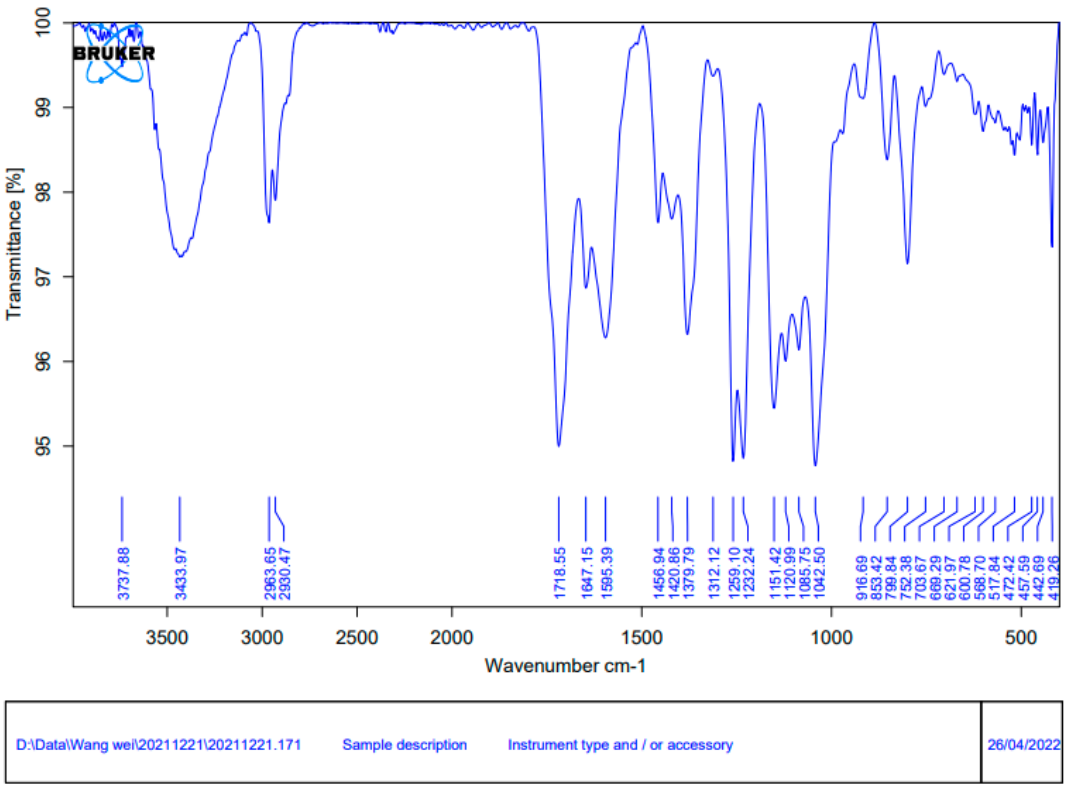

Figure S32. IR spectrum of **4**

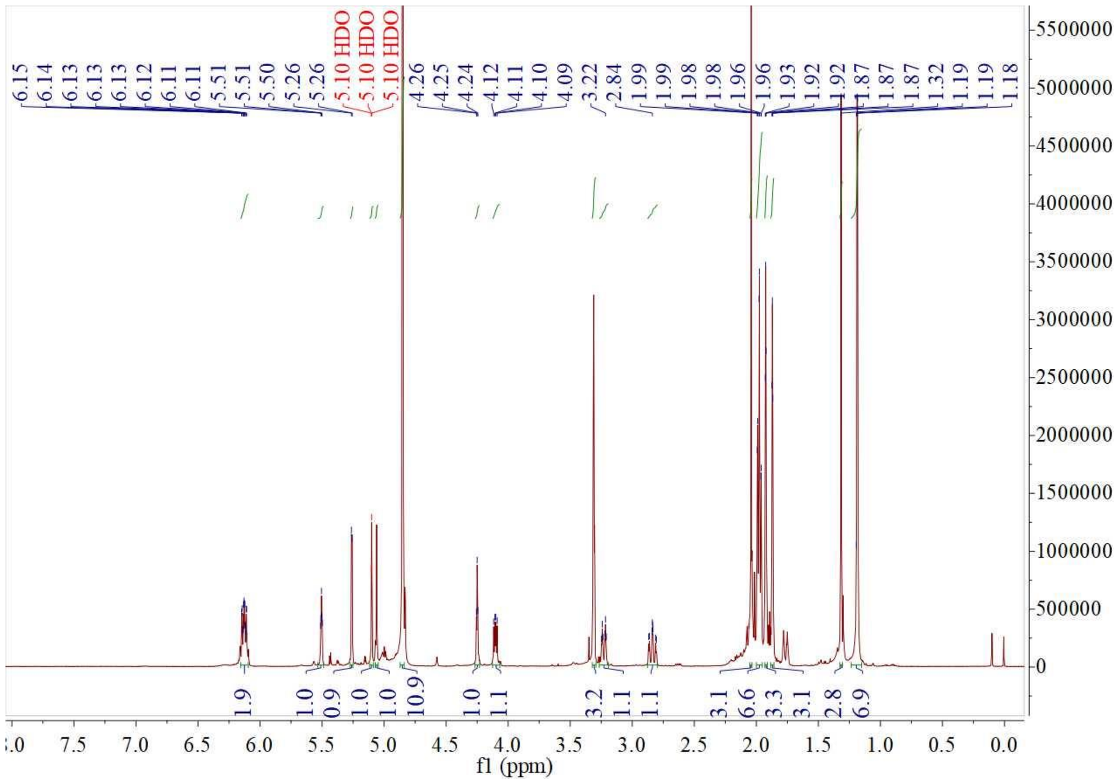

Figure S33. ^{1}H-NMR spectrum of **5** in MeOD (500 MHz).

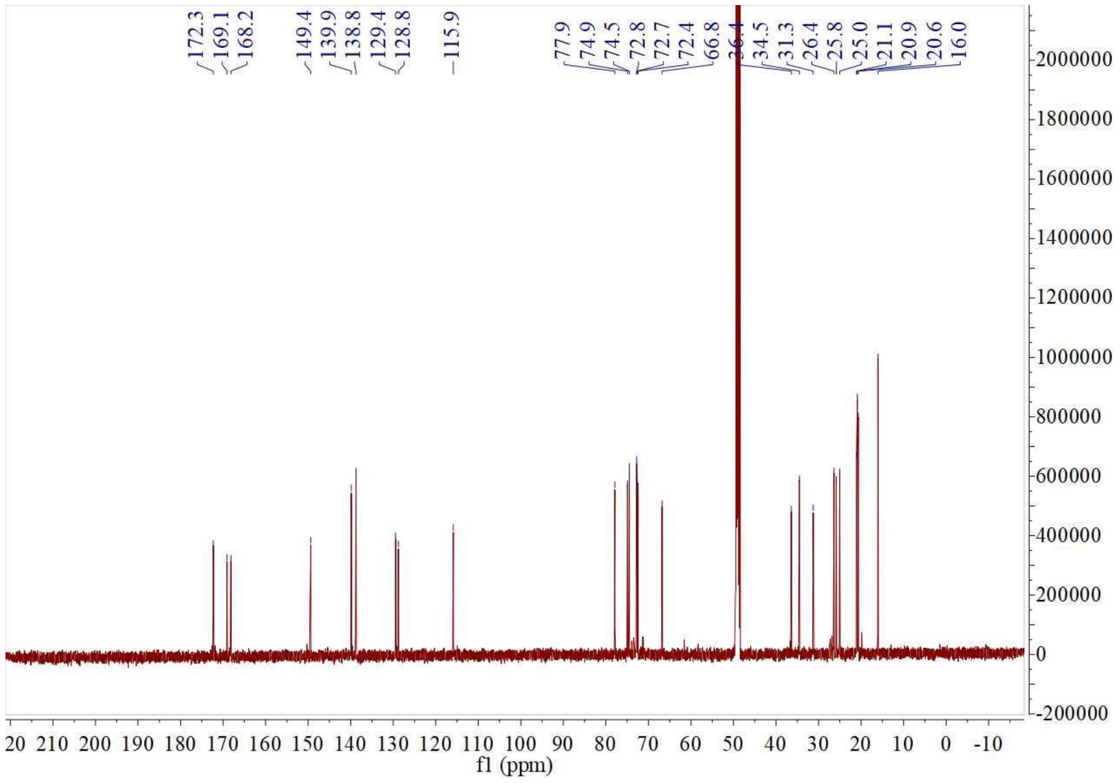

Figure S34. ^{13}C-NMR spectrum of **5** in MeOD (125 MHz)

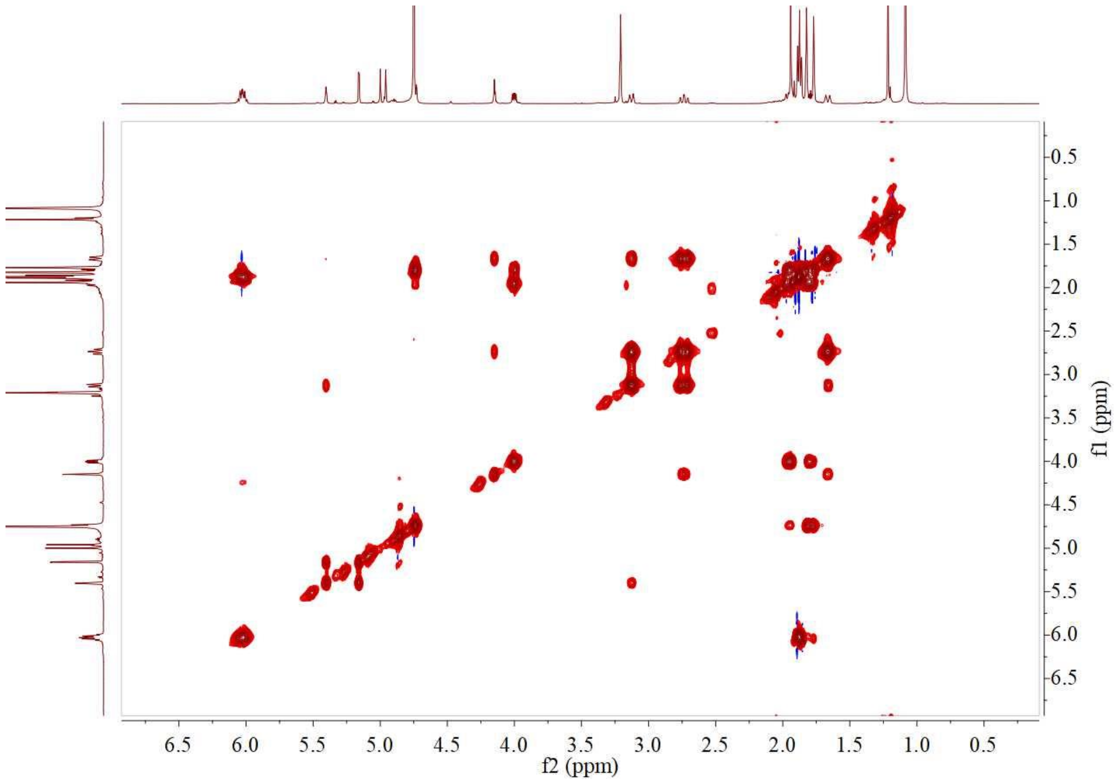

Figure S35. ^{1}H-^{1}H COSY spectrum of **5** in MeOD (500 MHz)

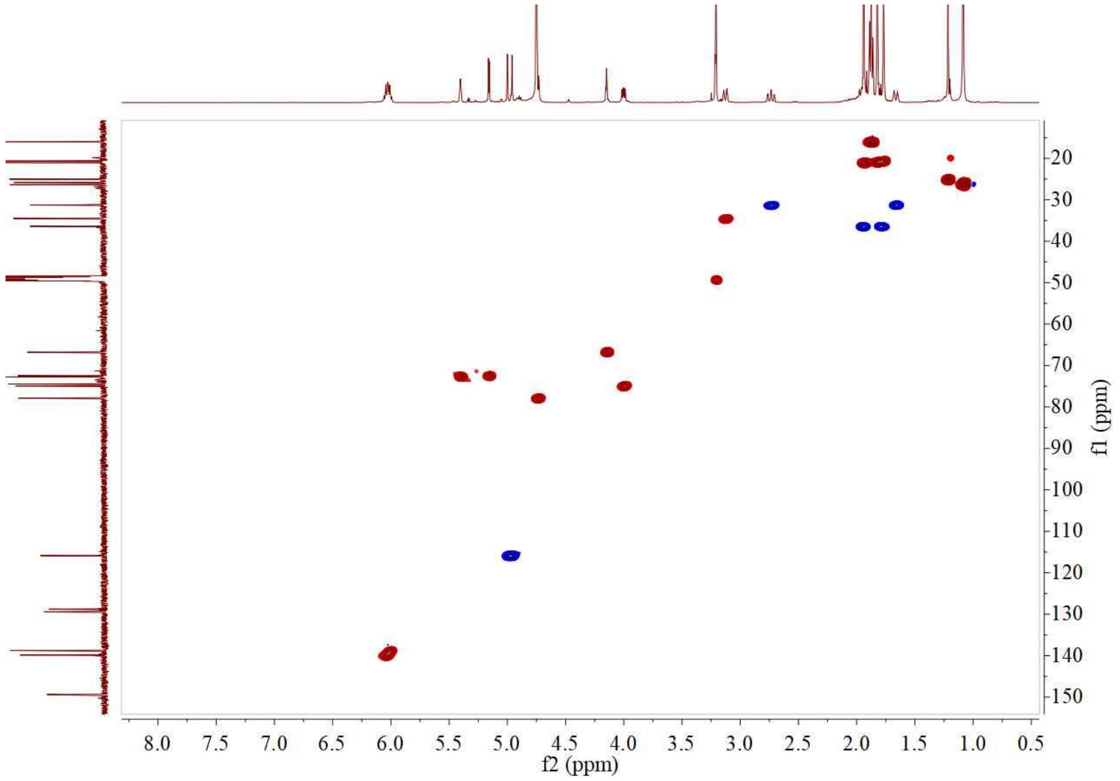

Figure S36. HSQC spectrum of **5** in MeOD (125 MHz).

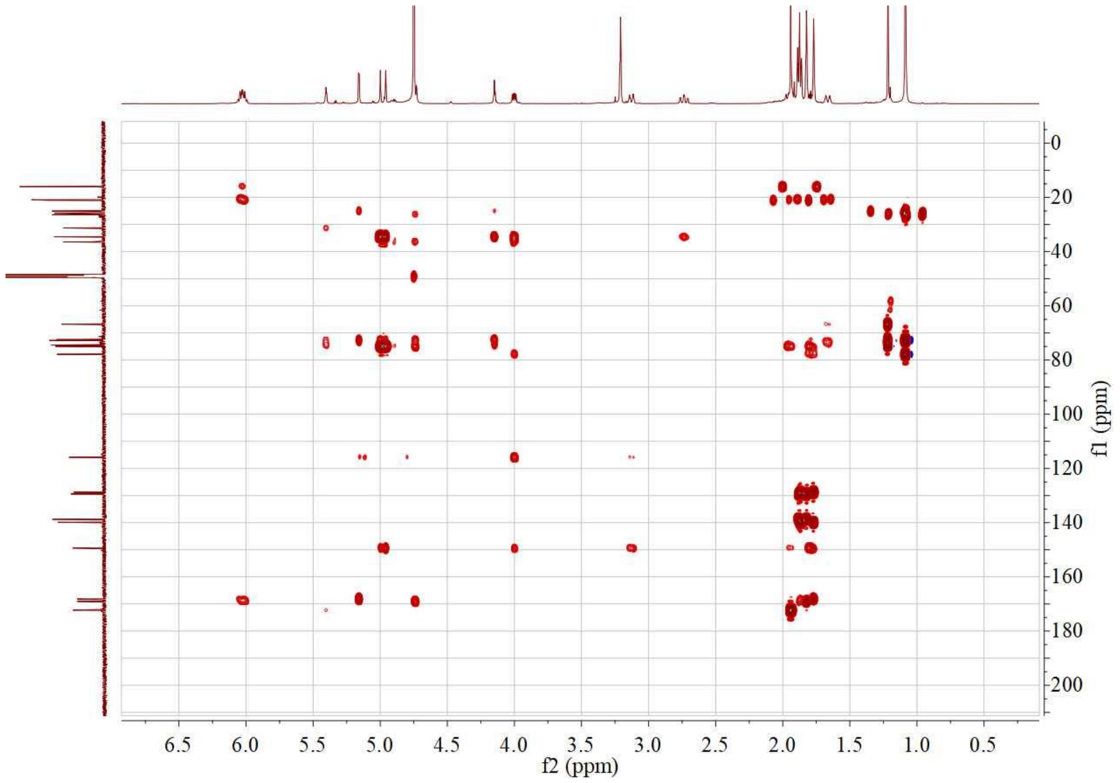

Figure S37. HMBC spectrum of **5** in MeOD (125 MHz).

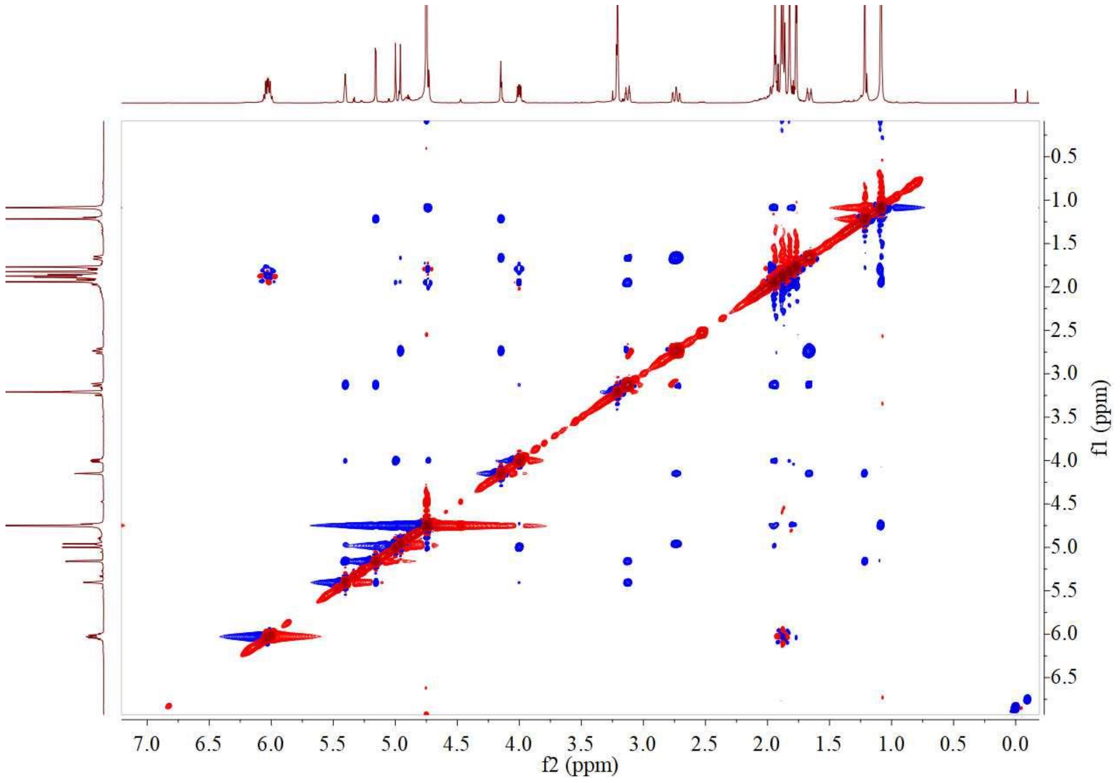

Figure S38. NOESY spectrum of **5** in MeOD (500 MHz).

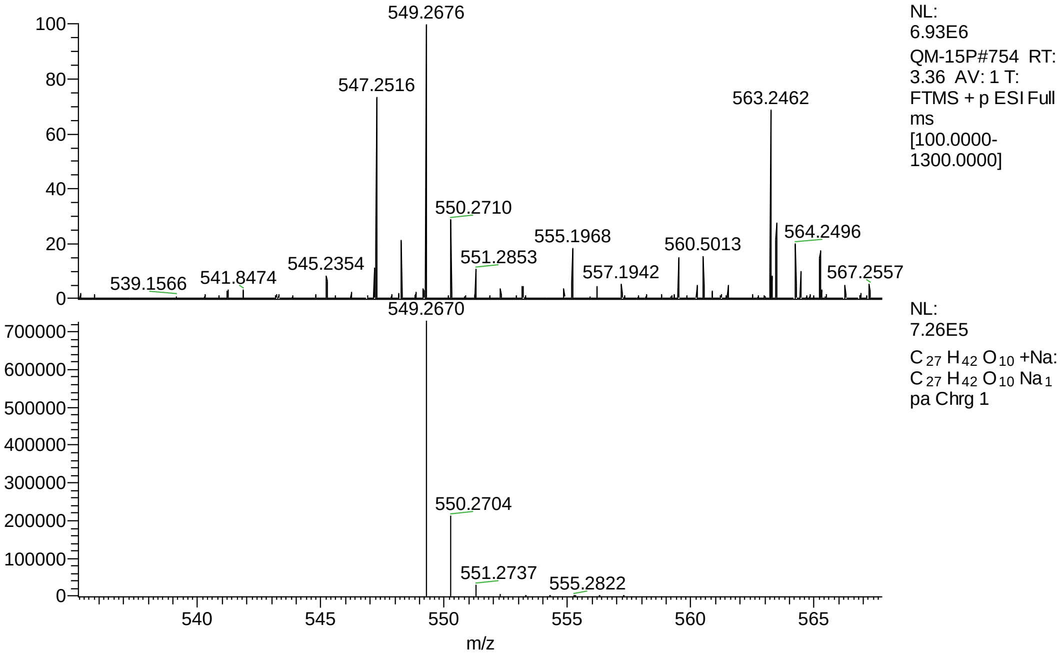

Figure S39. HR-ESI-MS spectrum of **5**

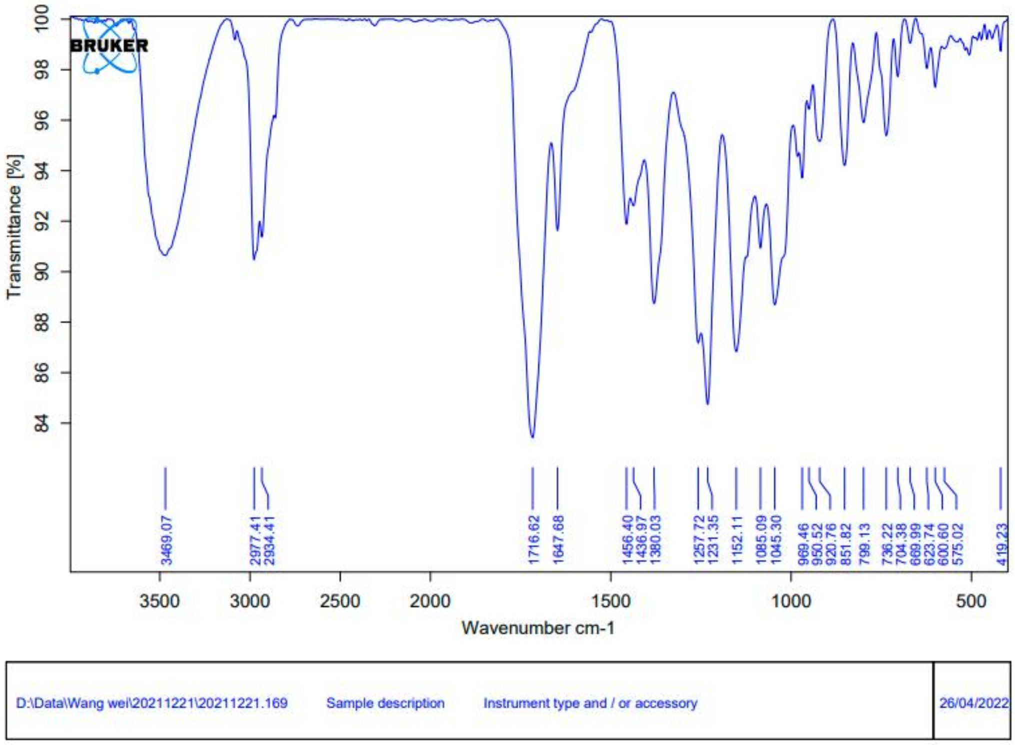

Figure S40. IR spectrum of **5**

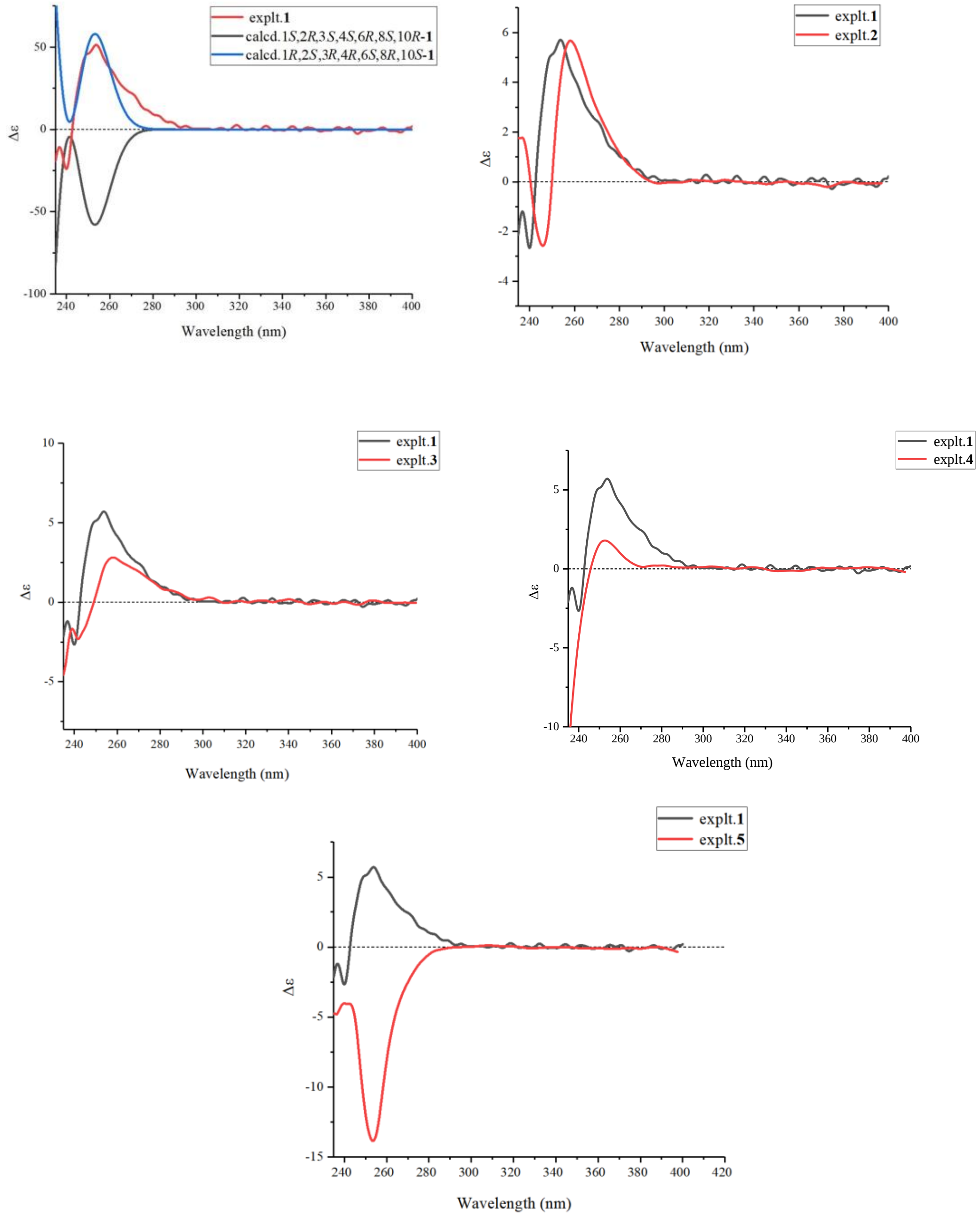

Figure S41. The ECD spectra of compounds **1-5**
